# Paneth Cells Regulate Lymphangiogenesis under Control of Microbial Signals during Experimental Portal Hypertension

**DOI:** 10.3390/biomedicines10071503

**Published:** 2022-06-25

**Authors:** Mohsin Hassan, Oriol Juanola, Irene Keller, Paolo Nanni, Witold Wolski, Sebastián Martínez-López, Esther Caparrós, Rubén Francés, Sheida Moghadamrad

**Affiliations:** 1 Department of Hepatology & Gastroenterology, Charité Universitätsmedizin Berlin, 13353 Berlin, Germany; mohsin.hassan@charite.de; 2 Department for Biomedical Research (DBMR), University of Bern, 3008 Bern, Switzerland; 3 Laboratories for Translational Research, Department of Gastroenterology and Hepatology, Ente Ospedaliero Cantonale, 6500 Bellinzona, Switzerland; oriol.juanola.juarez@usi.ch; 4 Faculty of Biomedical Sciences, Università della Svizzera Italiana, 6900 Lugano, Switzerland; 5 Interfaculty Bioinformatics Unit, Swiss Institute of Bioinformatics, University of Bern, 3008 Bern, Switzerland; i.keller@nexus.ethz.ch; 6 Functional Genomics Center Zurich, University/ETH Zurich, 8057 Zurich, Switzerland; paolo.nanni@fgcz.uzh.ch (P.N.); witold.wolski@fgcz.uzh.ch (W.W.); 7 Hepatic and Intestinal Immunobiology Group, Departamento Medicina Clínica, Universidad Miguel Hernández, 03550 Alicante, Spain; sebastian.martinez@goumh.umh.es (S.M.-L.); ecaparros@umh.es (E.C.); rfrances@umh.es (R.F.); 8 Instituto de Investigación Sanitaria ISABIAL, Hospital General Universitario, 03010 Alicante, Spain; 9 Instituto de Investigación, Desarrollo e Innovación en Biotecnología Sanitaria de Elche (IDiBE), Universidad Miguel Hernández, 03207 Elche, Spain; 10 CIBERehd, Instituto Salud Carlos III, 28029 Madrid, Spain; 11 University Clinic of Visceral Surgery and Medicine, Inselspital, 3008 Bern, Switzerland

**Keywords:** lymphatic vessels, lymphangiogenesis, LYVE1, Paneth cells, portal hypertension

## Abstract

Intestinal microbiota can modulate portal hypertension through the regulation of the intestinal vasculature. We have recently demonstrated that bacterial antigens activate Paneth cells (PCs) to secrete products that regulate angiogenesis and portal hypertension. In the present work we hypothesized that Paneth cells regulate the development of lymphatic vessels under the control of intestinal microbiota during experimental portal hypertension. We used a mouse model of inducible PCs depletion (Math1^Lox/Lox^VilCreER^T2^) and performed partial portal vein ligation (PPVL) to induce portal hypertension. After 14 days, we performed mRNA sequencing and evaluated the expression of specific lymphangiogenic genes in small intestinal tissue. Intestinal and mesenteric lymphatic vessels proliferation was assessed by immunohistochemistry. Intestinal organoids with or without PCs were exposed to pathogen-associated molecular patterns, and conditioned media (CM) was used to stimulate human lymphatic endothelial cells (LECs). The lymphangiogenic activity of stimulated LECs was assessed by tube formation and wound healing assays. Secretome analysis of CM was performed using label-free proteomics quantification methods. Intestinal immune cell infiltration was evaluated by immunohistochemistry. We observed that the intestinal gene expression pattern was altered by the absence of PCs only in portal hypertensive mice. We found a decreased expression of specific lymphangiogenic genes in the absence of PCs during portal hypertension, resulting in a reduced proliferation of intestinal and mesenteric lymphatic vessels as compared to controls. In vitro analyses demonstrated that lymphatic tube formation and endothelial wound healing responses were reduced significantly in LECs treated with CM from organoids without PCs. Secretome analyses of CM revealed that PCs secrete proteins that are involved in lipid metabolism, cell growth and proliferation. Additionally, intestinal macrophages infiltrated the ileal mucosa and submucosa of mice with and without Paneth cells in response to portal hypertension. Our results suggest that intestinal microbiota signals stimulate Paneth cells to secrete factors that modulate the intestinal and mesenteric lymphatic vessels network during experimental portal hypertension.

## 1. Introduction

The lymphatic system consists of lymph, lymphatic vessels and lymphoid organs that act as a parallel circulatory network to the blood vasculature [1]. Lymphatic capillaries are formed by a monolayer of lymphatic endothelial cells (LECs) connected by discontinuous button-like junctions facilitating the entrance of interstitial fluids, immune cells and macromolecules [2]. The biological functions of lymphatic vessels are the maintenance of tissue fluid homeostasis, tissue immunosurveillance, dietary fat absorption and transport in the digestive system [3]. The formation and maintenance of lymphatic vessels depend on several molecular factors such as cell surface lymphatic vessel endothelial hyaluronan receptor 1 (*LYVE1*), the transcription factor prospero-related homeobox 1 (*PROX1*), vascular endothelial growth factor C (*VEGFC*) and its tyrosine kinase receptor 3 (*VEGFR3*), among others [4].

An impairment in the lymphatic flow or structural changes of the lymphatic system may lead to severe clinical manifestations and has been reported in various pathological conditions such as inflammatory bowel disease, cancer, and chronic liver disease [5,6,7]. Advanced chronic liver disease (CLD) is associated with an increased density of lymphatic vessels and/or an increase in lymph flow because of portal hypertension [8,9,10]. Lymphatic imbalance characterized by insufficient splanchnic and peripheral lymph drainage has been described during CLD and proposed as a plausible origin of ascites formation [11,12]. In addition to the increased abundance of lymphatic vessels as a physiological compensation, the change in size of these vessels has been found in patients or animal models as a result of an increase in lymph production during advanced CLD [13,14]. While structural and functional changes in the lymphatic system have been studied in models of chronic liver disease, knowledge about the lymphatic network within the intestinal or mesenteric circulation and its function in the development of portal hypertension is limited [15]. 

We demonstrated in the past that intestinal microbiota contribute to the development of portal hypertension through regulation of intestinal blood and lymphatic vessels [16]. We observed that Paneth cells were also affected not only by the presence of intestinal microbiota, but also after experimental portal hypertension, suggesting a link between portal hypertension, intestinal vascularization and PCs [16]. PCs are members of intestinal innate immunity that control the quantity and diversity of intestinal microbiota [17], while hampering pathogen invasion [18] by releasing antimicrobial peptides, such as α-defensin, lysozyme, regenerating islet derived protein 3 (REG3A) and angiogenin 4. Recently, we have demonstrated that pathogen-associated molecular patterns (PAMPs) stimulate PCs to secrete pro-angiogenic factors which promote intestinal and mesenteric vascularization, thus contributing to increased experimental portal hypertension [19]. These results support previous studies describing the role of intestinal microbiota and PCs in modulation of intestinal angiogenesis [20,21]. Since the blood and lymphatic vessels are complementary circulatory systems, further studies aimed at better understanding the potential role of PCs in the regulation of lymphatic system are of the utmost interest. Therefore, we were interested in a more in-depth investigation to better understand the relationship between Paneth cells and their possible role in the regulation of lymphatic vessels.

Based on previous results and current knowledge about the role of intestinal microbiota and PCs in the development of intestinal vascularization, in the present study, we speculated that factors released by PCs may contribute to the regulation of intestinal and mesenteric lymphatic vasculature during experimental portal hypertension. 

## 2. Material and Methods

### 2.1. Animals

Nine-week-old male Math1^Lox/Lox^VilCreER^T2^ (hereafter designated as Math1^Δintestine^ or Math1^Δint^) mice with a mixed C57BL/6 and SV129 background were used for the study. Math1^lox/lox^VilCreER^T2^ and control (cre negative) littermates were housed under specific pathogen-free (SPF) conditions in a ventilated cage system with a 12 h light/dark cycle in the central animal facility of the University of Berne. The depletion of PCs was achieved by activation of Cre recombinase after injection of 1 mg/mice/day tamoxifen (Tm, Sigma T5648) for three consecutive days in Math1^Δintestine^ and control littermate mice [22]. Partial portal vein ligation (PPVL) was carried out 5 days after the last tamoxifen injection under sterile conditions to induce pre-hepatic portal hypertension, or sham-operated as described in the past [19]. Animals were euthanized and tissues were harvested 14 days after PPVL for further experiments. All animal experiments were performed according to the international regulations concerning conduct of animal experimentation. Experimental protocols were approved by research animal ethics committee of the canton of Bern (authorization number BE 94/16).

### 2.2. Genotyping of Mice

Ear biopsies from Math1^Δintestine^ and control mice were obtained and processed for the isolation of DNA using a DNA isolation kit (Macherey-Nagel GmbH, Düren, Germany). Following the manufacturer’s instructions, pure DNA was eluted and 10 µL of DNA (10 ng/µL) was used to detect Cre recombinase and Math-1. The primers used for genotyping are provided in Supplementary Table S1.

### 2.3. RNA Isolation 

Total RNA was purified from 50 mg distal intestine (ileum) of all experimental groups using the RNeasy Plus Mini Kit (Qiagen, Hilden, Germany). Following manufacturer’s instructions, the RNA extracts were eluted in 30 μL of RNase and DNase free water. The total yield of RNA was quantified using a nanodrop spectrophotometer (Thermofisher, Waltham, MA, USA).

### 2.4. mRNA-Sequencing in Small Intestinal Tissue 

Transcriptomic analysis (n = 4/group) was performed as previously described [19]. Briefly, the quality of RNA was evaluated using the Qubit RNA BR Assay kit (ThermoFisher, Q10211) and an advanced analytical fragment analyzer RNA kit (Agilent, Santa Clara, CA, USA, DNF-47). RNA libraries were prepared using an Illumina Truseq Stranded mRNA Library Prep kit (Illumina, Santa Clara, CA, USA, 20020595), following the manufacturer’s instructions, and libraries were sequenced paired-end (2 × 50 bp) using an Illumina NovaSeq 6000 sequencer. The next generation sequencing (NGS) reads were mapped to the reference genome of mouse (GRCm38.94) using HiSat2 v. 2.1.0, and the number of overlapping reads for each gene was counted with Feature Counts v. 1.6.0 [23,24]. Differential expression analysis was performed using DESeq2 v.1.24.0 in R v. 3.6.1 [25]. The Benjamini and Hochberg procedure [26] was used to calculate adjusted *p* values (*p*-adj), and genes with a *p*-adj < 0.05 were considered significant. Heatmaps were generated with gplots v. 3.0.4 in R v.3.6.1. The weight01 algorithm in topGO v. 2.38.1 was used for Gene Ontology enrichment analysis. Major terms with at least 2 genes were selected based on the *p*-values from a Fisher’s exact test.

### 2.5. RT^2^ Profiler qPCR Array and Gene Expression Quantification by Real-Time PCR

Reverse transcription was performed with M-MLV Reverse transcriptase (Invitrogen) and a random hexamer mix (PROMEGA, USA) for cDNA synthesis. RT^2^ SYBR Green qPCR master mix (Qiagen) was mixed with cDNA synthesis reaction as per defined protocol, and loaded using a multichannel pipette onto a customized qPCR plate. The plate was sealed with thin-walled caps provided with the kit. Before performing the qPCR reaction in a 7900 HT real time cycler (Thermofisher), the plate was briefly centrifuged for 1 min to ensure all the contents were settled at the bottom of the wells. The qPCR results were obtained using Mouse Angiogenesis RT^2^ Profiler PCR Array (96 well format; Qiagen). Then, 1 μL of cDNA was used for real-time PCR using *PROX1*, forkhead box C2 (*FOXC2*), *VEGFR2* and *VEGFR3* specific primers and probes (Supplementary Table S2), and TaqMan™ Fast Universal PCR Master Mix (ThermoFisher). Using the ΔΔC_T_ method, C_T_ values for each gene were subtracted from the endogenous control gene *GAPDH*, and then normalised to experimental control sham mice. All experiments were performed in triplicate (n = 5 each group).

### 2.6. Immunohistochemistry

Swiss rolls (≈ 5 cm) of tissue sections from distal (ileum) and proximal (duodenum) intestine were prepared and fixed in 10% buffered formalin solution for 24 h, as previously described (n = 5/group) [27]. After embedding in paraffin, 5-micron thick sections were prepared and mounted on a glass slide. The slides were heated at 45 °C for 15–20 min, incubated in xylol for 10 min to deparaffinize the tissue sections, and then rehydrated in graded ethanol. Slides were incubated for 10 min with 0.4% hydrogen peroxide (H_2_O_2_) and antigen retrieval was performed in sodium citrate buffer (pH 6) for 15 min. The tissue sections were blocked with TBS/1% BSA/0,1% Tween-20 for 1 h at room temperature to prevent non-specific binding. The sections were subsequently incubated overnight at 4 °C with anti-CD3 (1:250, Cell Signaling Technology, #78588), anti-F4/80 (1:200, Cell Signaling Technology, #70076), anti-LYVE1 (1:30, R&D, #BAF2125) and anti-lysozyme (1:500, Thermofisher, #PA5-16668) primary antibodies, followed by an incubation for 1 h at room temperature with biotinylated secondary goat anti-rabbit (1:400, Dako/Agilent, Santa Clara, CA, USA #E0433) or (1/200, Vector, #BA-1000). The sections were either treated with streptavidin, horseradish peroxidase or ABC solution, (Vector laboratories, Newark, CA, USA) for 30 min. Then, a mixed solution of 3,3-diaminobenzidine-tetrahydrochloride (DAB) substrate or AEC solution (Vector laboratories, Newark, CA, USA) was applied for color development of the reaction product, followed by hematoxylin counterstaining. A panoramic digital whole slide scanner (3D HISTECH Ltd., Budapest, Hungary) was used to scan the slides stained by immunohistochemistry. Ten different fields per slide were blindly photographed using CaseViewer 2.2 software (3D HISTECH Ltd., Budapest, Hungary). MetaMorph imaging software (NX software 64 bite, version 7.8.12.0) was used to quantify positive stained areas. The data is expressed as the total number of positive pixels divided by the total number of pixels (% area).

### 2.7. Isolation of Crypts

Small intestinal crypts were isolated from Math1^Δintestine^ and control animals, as described previously [28]. Briefly, the proximal part (duodenum) of the intestine of mice from each group was cut into two equal halves and washed twice in cold PBS + antibiotics (Penicillin, Streptomycin and Gentamycin). The tissue pieces were incubated with 15 mM ethylene diamine tetra acetic acid (EDTA) in PBS for 30 min on ice with discontinuous shaking. Afterwards, the tissue pieces were incubated with PBS + antibiotics and vortexed until supernatants were enriched with intestinal crypts. Each fraction obtained after vortexing was observed under the microscope. Crypt enriched fractions were filtered using 70 μm BD cell strainers and centrifuged at 300× *g* for 5 min at 4 °C. Pellets enriched in crypts were washed twice in 2 mL DMEM-F12 media (Dulbecco’s Modified Eagle Medium/Nutrient Mixture F-12), and then resuspended in Matrigel.

### 2.8. Development and Passaging of Intestinal Organoids

As described previously [28,29], around 1000 intestinal crypts resuspended in Matrigel were plated and incubated at 37 °C for 5 min to polymerize the Matrigel. Then, complete media (DMEM-F12) containing specific intestinal organoid growth factors R-Spondin 1 (500 ng/mL), EGF (50 ng/mL), Noggin (100 ng/mL), and Wnt3a (100 ng/mL), (Peprotech) supplemented with N2 and B27 (Invitrogen), were added, respectively. Fresh media was supplied every three days until day 9, until complete organoids were developed. Passaging of small intestinal organoids was performed by disrupting the 3D integrity of organoids in Matrigel at day 9 by adding 1 mL of pre-chilled DMEM-F12 + 1% HEPES to each well. Afterwards, the suspension was briefly triturated with a 1 mL pipette, and then collected in 15 mL falcon tubes and centrifuged at 1700× *g* for 5 min at 4 °C. The Matrigel and media in supernatant was carefully aspirated, and the pellet containing organoids was incubated with 0.48 mM EDTA in 1 PBS for 10 min with intermittent shaking to dissociate organoids into individual crypts. After 10 min, 2 mL of DMEM 10% FBS was added. The suspension was again centrifuged at 1700× *g* for 5 min at 4 °C. Afterwards, the supernatant was aspirated, the crypts were counted and resuspended in Matrigel, and then plated again at a 1:4 split ratio till desired experimental purpose was achieved.

### 2.9. Stimulation of Intestinal Organoids with PAMPs

After 9 days of culture, organoids were stimulated with PAMPs stimuli including lipopolysaccharide (LPS) (1 μg/mL; Sigma-Aldrich, St. Louis, MO, USA; from *E*. *coli* 026:B7), muramyl dipeptide (MDP) (100 μg/mL; InvivoGen), peptidoglycan (PGN) (10 μg/mL; InvivoGen, San Diego, CA, USA; from *S. aureus*) and CpG oligodeoxynucleotides (CpG ODN) 1668 (1 μg/mL; Enzo) for 24 h at 37 °C. After stimulation, organoids were washed twice with PBS and incubated with fresh media again for 48 h at 37 °C. Unstimulated organoids served as controls. The supernatants or conditioned media (CM) were collected after 24 h and stored at −80 °C for further analyses.

### 2.10. Whole Mount Lysozyme Immunofluorescence of Organoids and Imaging

Each 50 μL of Matrigel containing intestinal crypts isolated from Math1^Δintestine^ and control mice were plated in pre-warm chamber slides (SPL life sciences) and cultured under the culture conditions mentioned above. After 9 days of culture, the organoids were washed with warm PBS and fixed with 2% paraformaldehyde for 20 min. Subsequently, the organoids were washed with 1× PBS/glycine solution and permeabilized with 0.5% triton × 100 in PBS for 10 min on a shaker. The plates were blocked using 10% goat serum in wash buffer for 2 h. Primary antibody anti-lysozyme at 1:400 (Novus Biologicals, Littleton, CO, USA, #NBP2-33518) was applied for 4 h. Organoids were then incubated for 1 h at room temperature with the corresponding secondary antibody 1:500 (goat anti rabbit Alexa fluor 488, Invitrogen). The slides were then incubated with 4′,6′-diamidino-2-phenylindole (DAPI, 1:10,000) in 1× PBS and phalloidin at 1:500 for 10 min. Histokitt mounting medium (Carl Roth) was used to mount the slides. The imaging was performed to report presence or absence of PCs using a Leica DMI4000 B fluorescence system.

### 2.11. Lymphatic Endothelial Cell Tube Formation Assay

The capacity of dermal lymphatic microvascular endothelial cells (LECs) (Cat. No. XSEL6C1, Lonza, Basel, Switzerland) to form lymphatic tubes in vitro was assessed as previously described [19]. Briefly, LECs at passage 7 (20 × 10^3^/well) were starved for serum and growth factors overnight and seeded in 96-well plates coated with Matrigel using fresh endothelial cell growth basal medium-2 (Lonza). Cells were then washed with PBS and incubated with previously collected CM at 37 °C, 5% CO_2_. Micrographs were taken after 24 h using a digital camera integrated in an inverted light microscope (ZEISS Axio Vert.A1). All experiments were performed in triplicate. Auto contrast adjustment using PhotoScape X software (MOOII Tech, Seoul, Korea) was used to increase the contrast of all panels. The number of branched points where lymphatic vessels bifurcate per field area was counted manually to assess the formation of new tubes, as represented in Supplementary Figure S1.

### 2.12. Wound Healing Assay

LECs migration was evaluated in vitro as described before [19]. LECs at passage 7 (50 × 10^3^/well) were cultured in a 24-well plate until the cells were confluent, and then starved for serum and growth factors overnight. A scratch was made on the confluent cell monolayer using the narrow part of a 200 µL tip. Cells were then carefully washed with PBS and treated with organoid-derived CM at 37 °C, 5% CO_2_. Micrographs were taken at time 0 (T = 0) and after 12 h (T = 12) of incubation using a ZEISS Axio Vert.A1 microscope. All experiments were performed in triplicate. Auto contrast adjustment using PhotoScape X software was used to increase the contrast of all panels. The width of the scratch was measured using ImageJ software (Wayne Rasband, NIH, Bethesda, Maryland, USA) at T = 0 and T = 12 from 3 independent experiments. Wound recovery distance (µm) was calculated as the difference between the gaps at T = 0 and at T = 12.

### 2.13. Proteomic Analysis of Conditioned Media

Secretome analysis was performed as previously described [19]. Briefly, supernatants collected from LPS and MDP-treated Math1^Δintestine^ and control intestinal organoids were used for label-free proteomics quantification analysis using liquid chromatography–mass spectrometry (LC-MS) (n = 4/group). Proteins were identified and quantified using MaxQuant (version 1.6.2.3). Functions implemented in the R package SRMService [30] were run to compute *t*-tests among experimental groups (Math1^Δintestine^ vs. control) [31]. Detected proteins on each 2-group analysis were normalized by computing the z-score of log2 values. Proteins with at least 2 peptides and quantified with a false discovery rate < 1% were considered for further analysis. Proteins were ranked by log2 fold change, and gene set enrichment analysis (GSEA) was carried out using the gene ontology for biological processes functional database with the web-based gene set analysis toolkit (WebGestalt) [32]. Detailed proteomics analysis methods can be found in the Supplementary Methods.

### 2.14. Statistical Analysis

Statistical analyses were performed using GraphPad Prism software 5 (Software Inc., La Jolla, CA, USA). Data are reported as mean ± SD. Differences were considered significant at <0.05. Comparisons between two groups were performed using the Mann–Whitney U test, and more than two groups by Kruskal–Wallis one-way ANOVA.

## 3. Results

### 3.1. Paneth Cells Are Depleted in the Intestine of Math1^Lox/Lox^VilcreER^T2^ Mice after Tamoxifen Injection

**Figure 1:**
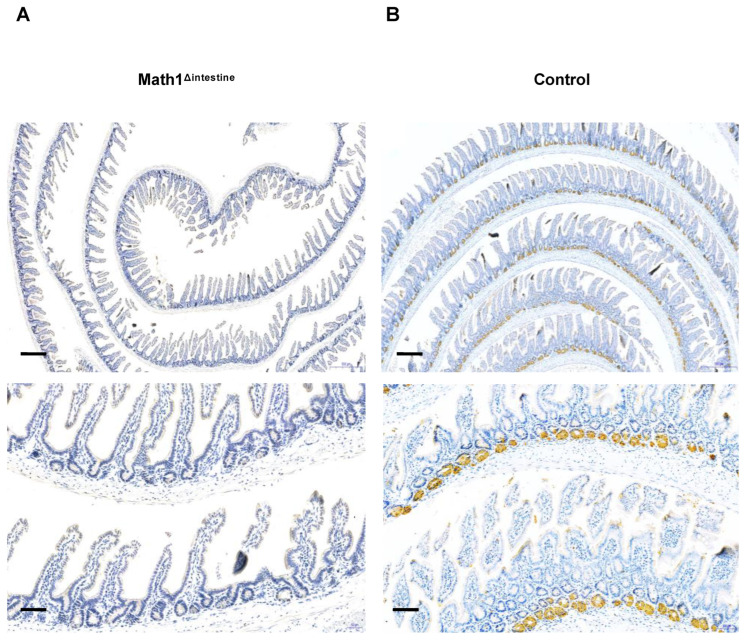
Intestinal phenotype of Math1^Δintestine^ and control mice after tamoxifen injection. Representative images of intestinal sections. The presence of PCs is observed by positive lysozyme immunostaining stained brown. (**A**) Small intestine of Math1^Δintestine^ mice and (**B**) small intestine of control mice. Black lines indicate 100 µm magnification (upper panels) or 50 µm magnification (lower panels), respectively. Abbreviations: Math1^Δintestine^, Paneth cell depleted; PCs, Paneth cells.

PCs secrete several antimicrobial peptides including lysozyme. Therefore, sections of the small intestine were stained with a lysozyme antibody by IHC to detect the presence or absence of PCs in our model (Figure 1). We observed that tamoxifen treatment induced a complete depletion of PCs in the small intestines of Math1^Δintestine^ animals (Figure 1A), whereas they remained in the base of intestinal crypts in control mice (Figure 1B).

### 3.2. Small Intestinal Gene Expression Is Altered in the Absence of Paneth Cells after Experimental Portal Hypertension

**Figure 2:**
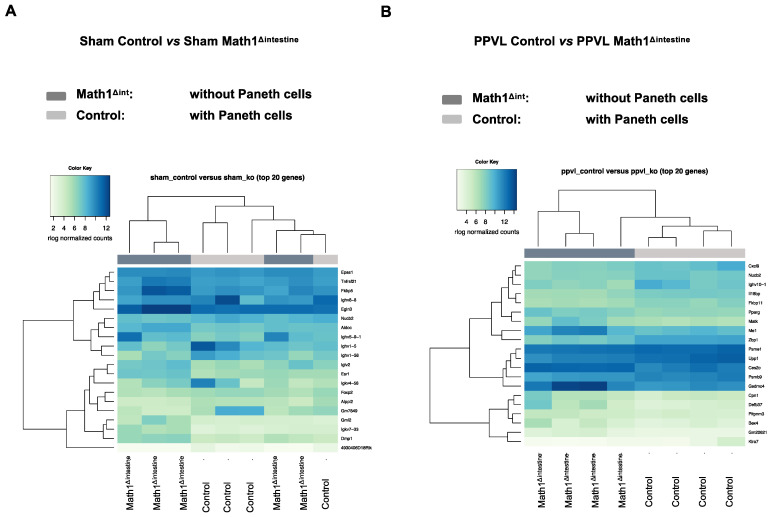
Paneth cells alter the intestinal gene expression pattern after induction of experimental portal hypertension. Gene expression analysis of small intestinal tissue of sham-operated and PPVL mice with or without PCs. Read counts are presented as heatmaps using the top 20 dysregulated genes (lowest *p*-adj values) in the small intestine of control and Math1^Δintestine^ under (**A**) basal conditions and (**B**) 14 days after PPVL. In the matrix view, the samples are represented in columns and the genes in rows. The color intensity represents the expression level, as shown in the legend. n = 4 per group. Abbreviations: Math1^Δintestine^, Paneth cell depleted; PCs, Paneth cells; PPVL, partial portal vein ligation; STRING, search tool for the retrieval of interacting genes/proteins; PPI, protein–protein interaction.

To study the effects of PCs depletion on gene expression signatures in the small intestinal tissue during experimental portal hypertension, we evaluated the gene expression profile in all experimental groups by mRNA sequencing (n = 4/group). We compared RNA-seq expression profiles between sham operated groups (control vs. Math1^Δint^) and after PPVL (control vs. Math1^Δint^). Detected genes were ranked based on ascending *p*-adj value; the top 20 are shown in Figure 2. We did not observe any distinct gene expression patterns in the small intestinal tissues of sham-operated animals regardless of the presence or absence of PCs (Figure 2A). We only found one differentially expressed gene associated with immune response (Immunoglobulin heavy variable V1-5, *Ighv1-5*) in sham-operated mice with PCs, as compared to the same conditions without PCs (*p*-adj < 0.05; Table 1). In contrast, animals with experimental portal hypertension showed differences in the gene expression pattern when comparing Paneth cell-deficient and control mice (Figure 2B), with 63 differentially expressed genes (*p*-adj < 0.05; Table 2).

Gene set enrichment analysis was performed for each comparison. While no significant GO terms were found among sham-operated conditions, several biological processes were enriched for genes differentially expressed between portal hypertensive mice with or without PCs (Table 3). Most of the biological processes affected by the absence of PCs during experimental portal hypertension were associated with immune-related responses, as expected. However, additional pathways relevant in lymphatic vessel functions including lipid metabolism (GO:0046321) and cell proliferation (GO:0042127) were also affected. Since previous data demonstrated that PCs are involved in the development of the intestinal vasculature [19,21], and biological functions associated to intestinal lymphatics vessels are affected in the mRNA sequencing analysis, we decided to further characterize whether PCs influence the intestinal lymphatic vasculature in the context of portal hypertension.

### 3.3. The Absence of Paneth Cells Is Associated with Reduced Expression of Lymphangiogenic Genes in Small Intestine of Portal Hypertensive Mice

**Figure 3:**
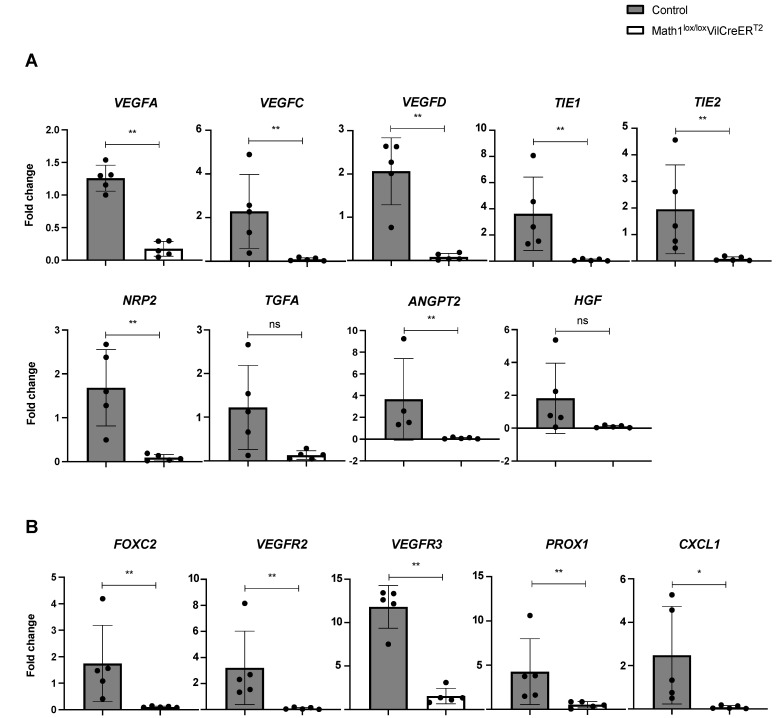
The expression of lymphangiogenic genes in the small intestine is affected by PCs in response to portal hypertension. Bar graphs showing (**A**) RT^2^ PCR array mRNA expression of lymphangiogenic genes in the intestine of PPVL-Math1^Δintestine^ vs. PPVL-control mice normalised to sham, and (**B**) RT-qPCR mRNA expression of specific lymphangiogenic genes in the intestine of PPVL-Math1^Δintestine^ vs. PPVL-control mice normalised to corresponding sham. Abbreviations: Math1^Δintestine^, Paneth cell depleted; PCs, Paneth cells; PPVL, partial portal vein ligation; vs, versus; *VEGFA*, *C*, *D*, vascular endothelial growth factor A, C, D; *NRP2*, neuropilin-2; TIE1, 2, tyrosine kinase with immunoglobulin-like and EGF-like domains 1, 2; *ANGPT2*, angiopoietin 2; *HGF*, hepatocyte growth factor; *TGFA*, transforming growth factor α; *FOXC2*, forkhead box protein C2; *VEGFR2*, *3*, vascular endothelial growth factor receptor 2, 3; *PROX1*, prospero homeobox 1; *CXCL1*, CXC motif chemokine ligand 1; ns, not significant. Data are expressed as mean ± SD. n = 5 per group. * *p* < 0.05; ** *p* < 0.005.

To confirm the results obtained from mRNA sequencing, we investigated whether the depletion of PCs could affect the mRNA expression of genes involved in proliferation and maintenance of lymphatic vessels using RT^2^ qPCR array and real-time qPCR (n = 5/group). The results from RT^2^ qPCR analysis revealed that *VEGFA*, *VEGFC*, *VEGFD*, *NRP2*, *TIE1*, *TIE2* and *ANGPT2* genes were significantly downregulated in Math1^Δintestine^ mice as compared to control mice after PPVL. *HGF* and *TGFA* were not significantly different between the two groups (Figure 3A). Consistently with RT^2^ qPCR analysis, we observed that the real-time mRNA expression of more specific markers of lymphangiogenesis, such as *PROX1*, *FOXC2*, *VEGFR2* and *VEGFR3* in Math1^Δintestine^ mice, was significantly diminished as compared to control mice after PPVL (Figure 3B).

### 3.4. Loss of Paneth Cells Is Associated with Reduced Abundance of Intestinal and Mesenteric Lymphatic Vessels after PPVL

**Figure 4:**
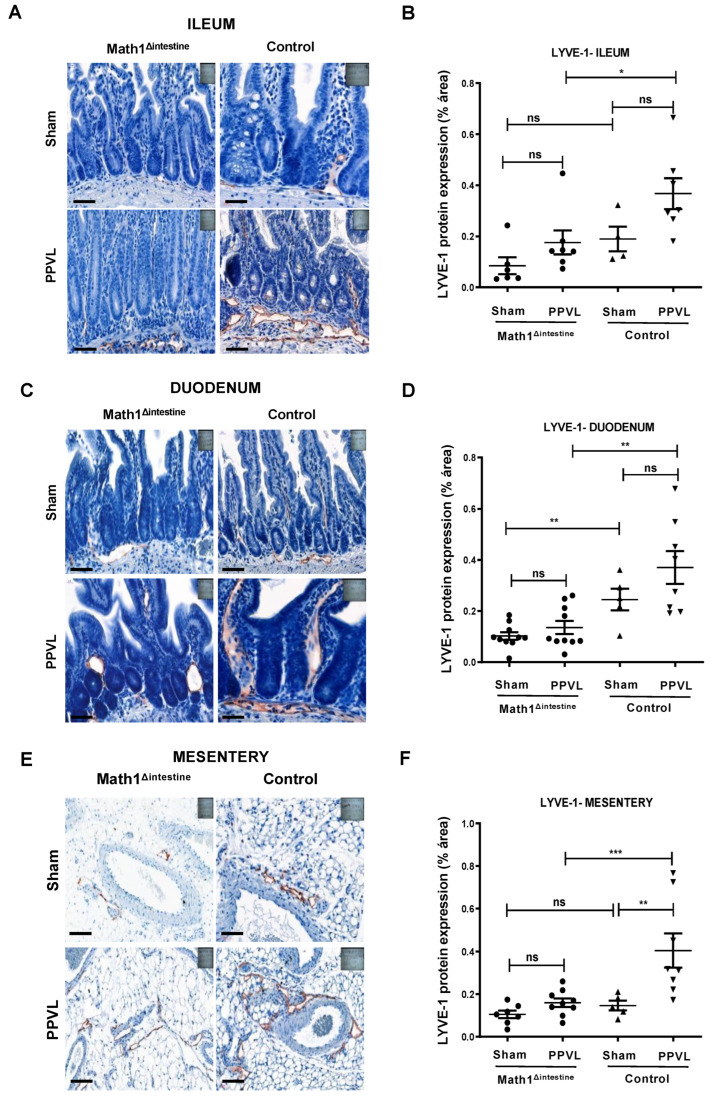
PCs modulate intestinal and mesenteric lymphangiogenesis. Representative immunostaining images and quantification showing the density of lymphatic vessels (stained brown) using LYVE1 antibody in sham-operated and portal hypertensive mice 14 days after PPVL in (**A**,**B**) distal intestine, (**C**,**D**) proximal intestine, (**E**,**F**) mesentery of Math1^Δintestine^ and control mice, respectively. Black line indicates 20 µm magnification. Abbreviations: Math1^Δintestine^, Paneth cell depleted; PPVL, partial portal vein ligation; LYVE1, lymphatic vessel endothelial hyaluronan receptor 1; ns, not significant. Data are expressed as mean ± SD. n = 5 per group. * *p* < 0.05; ** *p* < 0.005; *** *p* < 0.001.

The effect of PC depletion on the density of lymphatic vessels was examined by the positive staining for LYVE1 in different segments of the intestine and mesentery (n = 5/group) (Figure 4). We observed that the absence of PCs was associated with reduced density of lymphatic vessels among PPVL groups in the ileum 0.176% ± 0.12 vs. 0.367% ± 0.15, (*p* = 0.011) (Figure 4A,B), duodenum 0.135% ± 0.08 vs. 0.370% ± 0.17, (*p* = 0.003), (Figure 4C,D) and mesentery 0.160% ± 0.06 vs. 0.404% ± 0.20, (*p* = 0.001) (Figure 4E,F), as compared to control mice. Additionally, the induction of experimental portal hypertension was associated with an increase in the abundance of mesenteric lymphatic vessels as compared to the sham-operated animals only in the presence of PCs 0.144 ± 0.12 vs. 0.404% ± 0.20 (*p* = 0.04) (Figure 4E,F).

### 3.5. The Growth of Intestinal Organoids Is Not Affected by the Absence of Paneth Cells

**Figure 5:**
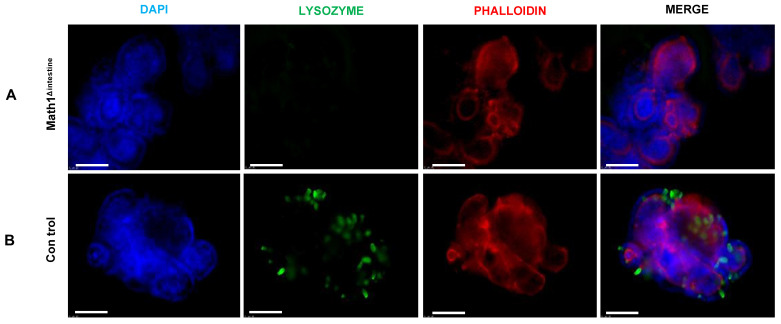
Intestinal organoids with or without PCs from control and Math1^Δintestine^ mice after tamoxifen injection. Representative lysozyme immunofluorescence images of organoids showing (**A**) absence of PCs in organoids isolated from Math1^Δintestine^ small intestine, and (**B**) presence of PCs (green) in organoids isolated from the intestinal crypts of control mice. Abbreviations: Math1^Δintestine^, Paneth cell depleted; PCs, Paneth cells.

To better understand the underlying mechanisms, we took advantages of culturing intestinal organoids isolated from the small intestinal crypts of control and Math1^Δintestine^ mice. We first confirmed the absence of PCs in the intestinal organoids developed from Math1^Δintestine^ mice by immunofluorescence using a lysozyme antibody. In organoids derived from control small intestine, the green fluorescence confirms the presence of lysozyme positive PCs (Figure 5).

### 3.6. PCs Secretory Products Stimulate Lymphangiogenic Responses In Vitro

**Figure 6:**
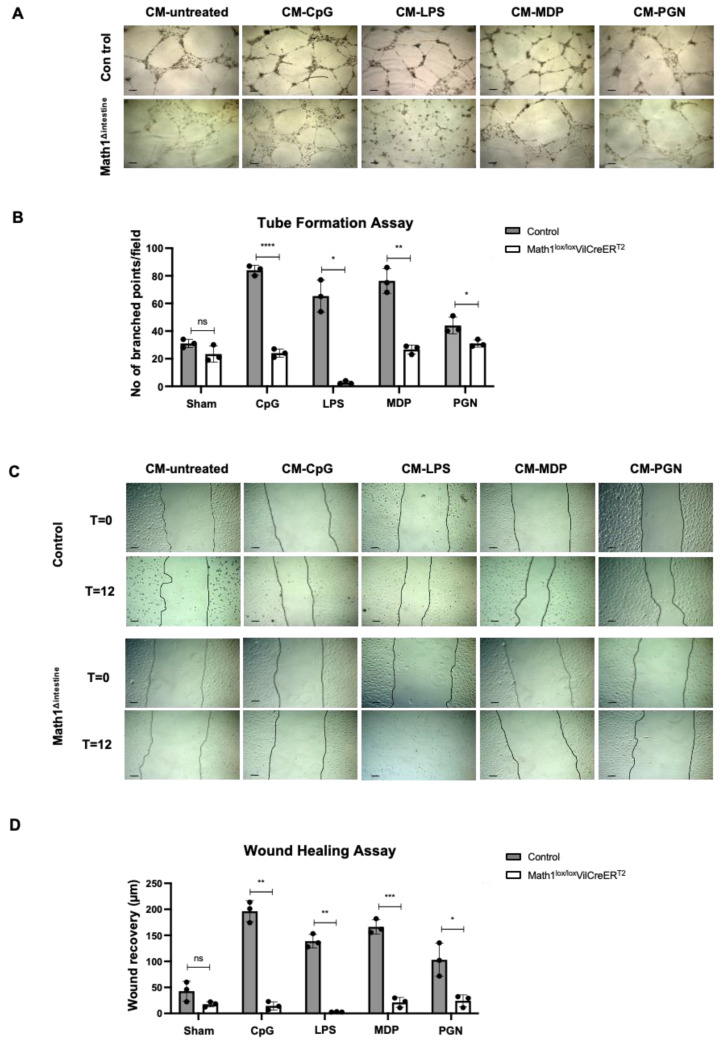
Lymphangiogenic properties of factors released by PCs in response to PAMPs. (**A**) Representative images showing tube formation assays of LECs co-cultured with CM for 24 h, collected from intestinal organoids unstimulated or stimulated with indicated microbial products in the presence or absence of PCs. Black lines indicate 100 µm magnification. (**B**) Quantification of tube formation assays indicating the number of branched points per field area. (**C**) Representative images showing wound healing assays of LECs co-cultured with CM collected from intestinal organoids unstimulated or stimulated with indicated microbial products in the presence or absence of PCs at time 0 h (T = 0) or after 12 h (T = 12) of incubation. Black lines indicate 50 µm magnification. (**D**) Quantification of wound healing assays indicating the distance (µm) that LECs migrated after 12 h (wound recovery). Abbreviations: CM, conditioned media; CpG, CpG motifs of bacterial DNA; LECs, lymphatic endothelial cells; LPS, lipopolysaccharide; Math1^lox/lox^ VilCreER^T2^, Paneth cell depleted; MDP, muramyl dipeptide; ns, not significant; PGN, peptidoglycan; PCs, Paneth cells. Data are expressed as mean ± SD and are representative of 3 independent experiments. * *p* < 0.05; ** *p* < 0.005; *** *p* < 0.001; **** *p* < 0.0001.

To confirm the role of PCs in the regulation of lymphangiogenesis in vitro, we stimulated PCs by challenging the intestinal organoids with PAMPs (CpG, LPS, MDP and PGN) for 24 h. The CM from unstimulated organoids or those challenged with PAMPs were collected. Lymphatic endothelial cells (LECs) were exposed to CM to evaluate whether PC-secreted factors in CM might induce lymphangiogenic activity in vitro. LECs treated with CM obtained from unstimulated organoids showed similar tube formation activities regardless of the presence or absence of PCs. However, the tube formation activity of LECs was significantly increased after treatment with CM derived from stimulated organoids with PCs, as compared to the same conditions in the absence of PCs (Figure 6A,B).

Lymphangiogenic activities of peptides secreted by PCs were further confirmed by performing wound scratch assays in LECs treated with the same CM, as discussed above. LECs migration remained unaltered when incubated with CM obtained from unstimulated organoids with or without PCs. Consistent with the results obtained in the tube formation assays, treatment of LECs with CM derived from PAMPs-challenged control organoids resulted in a significant increase of lymphatic cell migration, as compared to the same conditions in the absence of PCs (Figure 6C,D). Furthermore, we detected increased LECs proliferation after treatment with CM collected from PAMPs-challenged organoids with PCs (Supplementary Figure S2). These data are in line with the augmented haptotaxis and differentiation capacity of LECs treated under similar conditions in the presence of PCs. Collectively, these results suggest that factors secreted by PCs in response to bacterial products have lymphangiogenic properties in vitro.

### 3.7. Proteins Secreted by Paneth Cells Are Associated with Metabolic Processes, Cell Proliferation and Cell Growth

**Figure 7:**
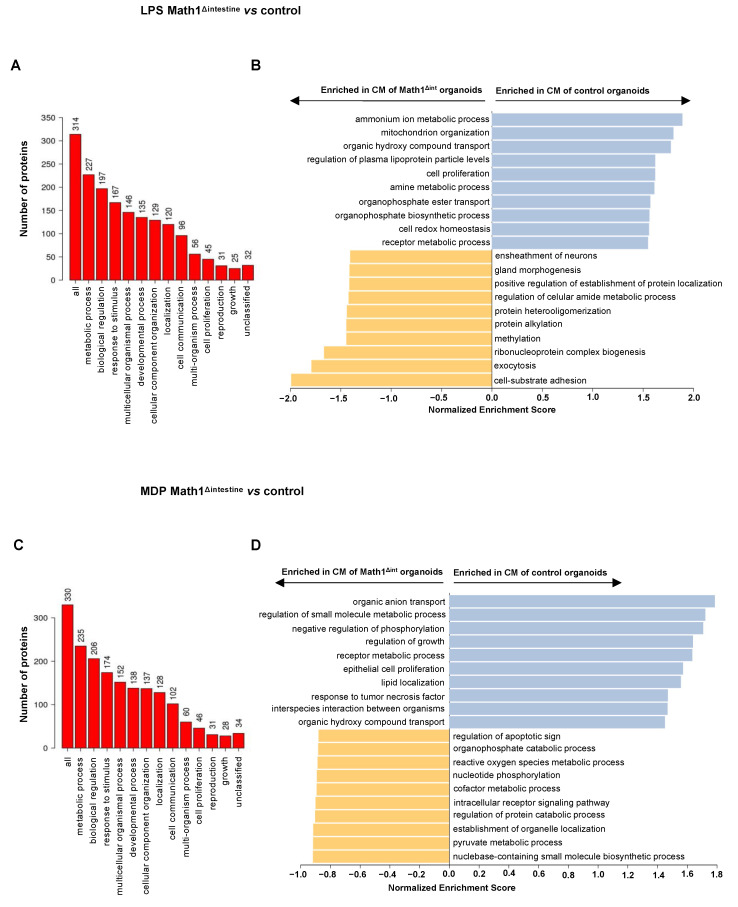
**PAMPs stimulate PCs to secrete factors associated with cell growth.** Secretome analysis of CM collected from LPS- and MDP-stimulated organoids with or without PCs. (**A**) GO slim bar chart of biological processes of the 314 proteins quantified in LPS-stimulated Math1^Δintestine^ vs. control organoids. (**B**) Bar chart showing GSEA of the 314 proteins quantified in LPS-stimulated Math1^Δintestine^ vs. control organoids. (**C**) GO slim bar chart of biological processes of the 330 proteins quantified in MDP-stimulated control organoids vs. Math1^Δintestine^ organoids. (**D**) Bar chart showing GSEA of the 330 proteins quantified in MDP-stimulated Math1^Δintestine^ vs. control organoids. The height of the bar represents the number of IDs in the user list and in the category in (**A**,**C**). Blue bars show positively, and yellow bars represent negatively enriched biological processes among identified proteins in (**B**,**D**). n = 4 per group. Abbreviations: GO, gene ontology; GSEA, gene set enrichment analysis; LPS, lipopolysaccharide; Math1^Δintestine^, Paneth cell depleted; MDP, muramyl dipeptide.

To validate whether PC-secreted products modulate the intestinal lymphatic network, we performed two-group analysis to compare differentially secreted proteins in CM after challenge with PAMPs (LPS and MDP) collected from intestinal organoids of control and Math1^Δintestine^ mice. We identified 314 proteins in the supernatants of LPS-challenged Math1^Δintestine^ vs. control organoids. The functions of these proteins were associated with metabolic processes, biological regulation, response to stimulus, cell communication, cell proliferation and cell growth, among others (Figure 7A). Additionally, gene ontology enrichment analysis (GSEA) revealed that biological processes associated with regulation of plasma lipoprotein particle levels, cell proliferation and cell redox homeostasis were enriched among the proteins secreted by control organoids challenged with LPS, while cell-substrate adhesion, protein metabolism and exocytosis were enriched in the factors secreted by organoids without PCs in response to this bacterial product (Figure 7B).

Similarly, secretome analysis performed in the supernatants of MDP-challenged Math1^Δintestine^ vs. control organoids quantified 330 proteins associated with metabolic processes, biological regulation, response to stimulus, cell proliferation and growth (Figure 7C). More specifically, regulation of growth, epithelial cell proliferation and lipid localization were biological processes enriched among the proteins secreted by PCs in control organoids after challenge with MDP, whereas regulation of protein catabolic process, nucleotide phosphorylation, reactive oxygen species, apoptotic pathway and cofactor metabolic process were biological processes enriched in the CM collected from organoids without PCs (Figure 7D). Taken together, these results confirm that PCs secrete proteins in response to PAMPs stimuli, which are involved in lipid metabolism, cell proliferation and growth. The complete list of the quantified proteins in both conditions is provided in Supplementary Table S3.

### 3.8. Intestinal Immune Cell Infiltration Is Slightly Affected by the Presence or Absence of Paneth Cells

**Figure 8:**
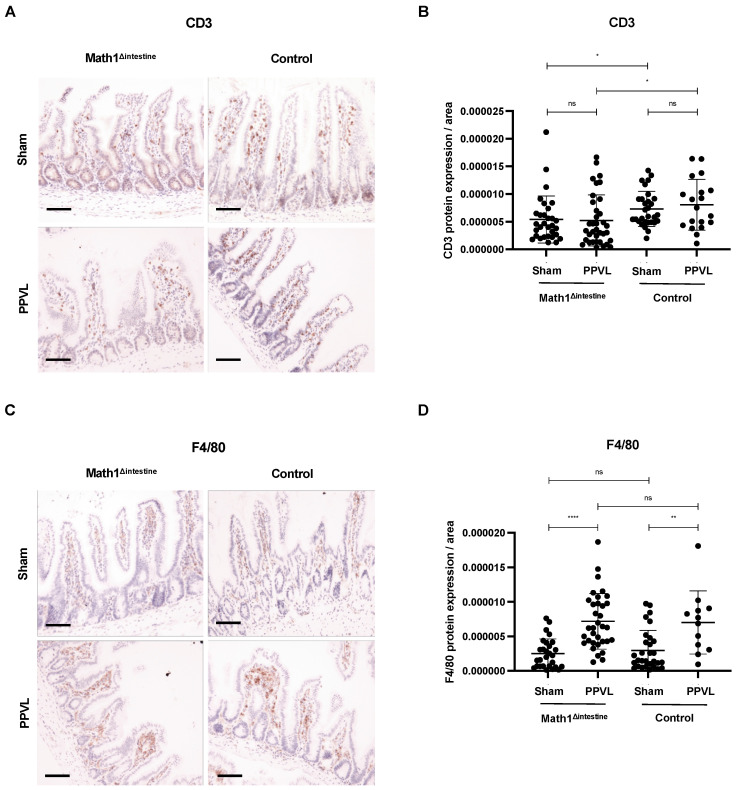
Intestinal immune cell composition. Representative immunostaining images showing intestinal T lymphocytes (**A**,**B**) and macrophages (**C**,**D**) in ileum tissue sections from the different study groups. Black line indicates 20 µm magnification. Abbreviations: Math1^Δintestine^, Paneth cell depleted; PPVL, partial portal vein ligation; CD3, cluster of differentiation 3; ns, not significant. Data are expressed as mean ± SD. n = 3–5 per group. * *p* < 0.05; ** *p* < 0.005; **** *p* < 0.0001.

We showed that secretory proteins from Paneth cells induce lymphangiogenic responses in portal hypertensive mice in vivo and in LECs in vitro. However, we sought to determine whether other factors may influence lymphatic vessels during portal hypertension in our model. It is known that lymphangiogenic factors can be released from different cell types, including fibroblasts, immune, tumoral and epithelial cells [33]. Since we detected reduced gene expression of *CXCL1* in the intestines of portal hypertensive mice without PCs (Figure 3B), we wondered whether PCs depletion could change the intestinal cell infiltration and recruitment of other immune cells. We evaluated, through immunohistochemistry, the expression of CD3 and F4/80 in ileal samples to detect the intestinal infiltration of lymphocytes and macrophages in our study groups. Among animals with the same genotype, the induction of experimental portal hypertension was not associated with a significant increase of lymphocyte infiltration as compared to sham-operated animals (Figure 8A,B). However, the ileum of mice with PCs presented a discrete increase in the infiltration of intestinal lymphocytes that reached significancy, as compared to the mice without PCs, independently of the induction of portal hypertension (Figure 8A,B). The infiltration of intestinal macrophages was significantly increased in the ileal mucosa and submucosa of portal hypertensive mice, as compared to sham-operated animals, independently of the presence or absence of PCs (Figure 8C,D).

## 4. Discussion

**Figure 9:**
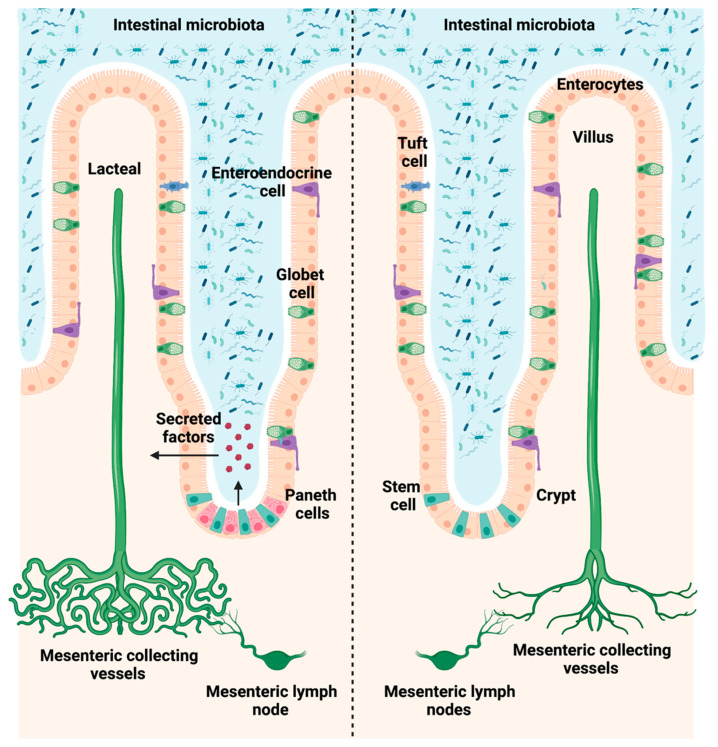
Paneth cells favor intestinal lymphangiogenic responses during portal hypertension.

In the present study, we demonstrated that PCs regulate the intestinal and mesenteric lymphatic proliferation during portal hypertension by modulating the density of lymphatic vessels and the expression of lymphangiogenic genes, as summarized in Figure 9. Our findings also provide evidence that bacterial products stimulate PCs to secrete pro-lymphangiogenic factors that may be involved in these processes.

Portal hypertension is associated with an increase in the splanchnic and hepatic lymphatic vessel density and lymph flow in chronic liver disease [34,35]. These results are in line with our previous observations showing increased lymphangiogenesis after PPVL [16]. In the absence of intestinal microbiota, we observed that portal hypertension, the density of intestinal lymphatic vessels and the number of PCs were significantly reduced. Moreover, in a recent study, our group showed the role of PCs as contributors to the intestinal vascularization and subsequent effects in portal hypertension [19]. Our current findings provide concrete evidence for the first time that intestinal and mesenteric lymphangiogenesis could also be under the control of PCs through microbial stimuli, in addition to portal hypertension.

Paneth cells secrete antimicrobial peptides in response to PAMPs that protect the intestinal milieu and prevent pathogen invasion [36]. Therefore, their activity is required to promote intestinal homeostasis and foster a balanced host–microbiota interaction. Indeed, Wehkamp et al. demonstrated that ileal Crohn’s disease is characterized by a compromised activity of PCs due to reduced production of alpha defensins [37]. The relevance of PCs in the pathogenesis of chronic liver disease was previously addressed in the study by Teltschik et al., where the authors demonstrated that reduced function of PCs predispose to bacterial translocation [38]. The regulatory role of PCs during development of portal hypertension was first evidenced in our work by performing mRNA sequencing of the small intestine. Unsupervised hierarchical clustering revealed that samples with different phenotypes for PCs showed similar gene expression patterns under basal conditions. The low number of differentially expressed genes under baseline might be due to the increased variability observed among the samples from mice without PCs. Nevertheless, following the induction of experimental portal hypertension, the gene expression pattern in the small intestinal tissue was significantly affected by the absence of PCs.

Multiple studies have previously reported the expression of specific markers to distinguish the lymphatic vessels from blood vessels [8,39,40,41]. We therefore evaluated the expression levels of *PROX1*, *FOXC2*, *VEGFC* and *VEGFR3*, and found a significant downregulation in portal hypertensive mice without PCs. These genes are required for the stimulation of lymphatic endothelial cells, as well as for the growth and maintenance of lymphatic vessels [42,43]. Therefore, our results suggest an association between PCs and the regulation of lymphatic vascular proliferation in the intestinal and mesenteric vascular beds during portal hypertension.

Lymphatic vessel endothelial hyaluronic acid receptor (LYVE) 1 is a binding membrane glycoprotein expressed on lymphatic endothelial cells [39]. Previously, increased intestinal expression of LYVE1 was reported in experimental models of portal hypertension [16]. We observed herein that PCs can affect the lymphatic vasculature since the absence of this cell population was associated with reduced intestinal and mesenteric lymphatic vessels, determined by LYVE-1 immunostaining, in portal hypertensive mice. Previous studies suggested a dysfunctional lymphatic drainage in advanced CLD and portal hypertension in patients and experimental models [12]. Fluorescent lymphangiography in cirrhotic rats revealed that the dysfunctional lymphatic drainage observed in peripheral, as well as splanchnic, circulation was associated with a high expression of endothelial nitric oxid synthase (eNOS) in mesenteric LECs [10,44]. Despite these findings, the precise cellular and molecular mechanisms explaining the role of the intestinal–mesenteric lymphatic system in the context of portal hypertension remains not fully understood.

Previous studies reported that intestinal microbiota is a major stimulus for PCs to regulate intestinal angiogenesis [19,21]. We further investigated the role of intestinal microbiota and its association with PCs for the regulation of lymphangiogenesis during portal hypertension. We observed that factors secreted by PCs in response to microbial products induced lymphangiogenic activities in LECs, as observed in tube formation and wound healing assays. Moreover, the gene set enrichment analysis performed after two-group proteomic analysis (Math1^Δintestine^ vs. control) revealed that proteins secreted by PCs following PAMPs-challenge were associated with lipid metabolism, cell proliferation and growth, among other biological pathways. LPS is a well-known pathogen-associated molecular pattern (PAMP) that elicits inflammatory responses. It activates and upregulates the secretion of cytokines and adhesion molecules, as well as inducing apoptotic cell death in endothelial cells [45]. Antimicrobial peptides secreted by PCs can recognize LPS and neutralize its cytotoxic activity in LECs [46]. Therefore, reduced antimicrobial peptides in the CM isolated from LPS-treated organoids without PCs might explain the deleterious effect of this CM on LECs. Collectively, these data supported our initial hypothesis that microbial derived signals may stimulate PCs to secrete factors with a potential role in the regulation of intestinal and mesenteric lymphangiogenic responses during the development of portal hypertension.

Different cell subtypes can influence the formation of lymphatic vessels by secreting lymphangiogenic molecules as VEGFC, VEGFD or Ang1. It is well known that infiltrating macrophages induce lymphangiogenesis [47], and their depletion in the cornea results in inhibition of lymphangiogenesis associated with reduced production of VEGFA, VEGFC and VEGFD [48]. Interestingly, the interaction between T lymphocytes and macrophages activates macrophages to secrete VEGFC and induce lymphangiogenesis [49]. Along these lines, removal of CD4^+^ using a specific anti-CD4 results in reduced expression of VEGFC, VEGFD and ameliorated lymphangiogenesis, as evidenced by reduced expression of Lyve-1 in the diapragm [50]. We observed compromised immune responses to portal hypertension in Math1^Δintestine^ mice, as shown by reduced ileal gene expression of the chemokine *CXCL1* that correlated with decreased infiltration of lymphocytes in the mucosa and submucosa of the ileum in sham and portal hypertensive Math1^Δintestine^ mice. It is possible that the small intestines of Math1^Δintestine^ mice are not fully immunocompetent and their capacity to respond to the intestinal stress induced by the congestion of the portal vein is altered. Although we can not exclude that other cellular sources can modulate the intestinal lymphatic vasculature, our data presented here suggest that secretory products of PCs may play an important role in this process.

We acknowledge that additional studies are required to identify the specific factors responsible for the lymphangiogenic effects of PC secretory products. It is important to note that the mRNA expression data is obtained directly from ileal tissue, while proteomics was performed in the supernatants obtained from organoids isolated from the small intestine. Since the expression of genes, and hence of proteins, is highly context-dependent, we should be cautious when comparing both datasets to identify molecular targets. Additionaly, we cannot exclude the possible effect of different multicellular responses in the presence or absence of PCs. Thus, the direct or indirect involvement of PCs in lymphangiogenesis needs to be elucitated in detail in the future.

## 5. Conclusions

In conclusion, our results suggest that PCs actively promote intestinal and mesenteric lymphangiogenesis in a process regulated by intestinal microbiota in experimental portal hypertension. These findings represent novel significant knowledge in the understanding of portal hypertension, suggesting that the components of the innate immune system (i.e., Paneth cells) are also involved in the regulation of previously unrelated mechanisms (i.e., portal pressure). This study supports further investigations to address the potential benefits of targeting the secretory products of Paneth cells as a new therapeutic intervention to diminish portal pressures in portal hypertension.

## Figures and Tables

**Table 1:** Differentially expressed gene detected in the comparison of sham control vs. sham Math1^Δint^.

Sham Control vs. Sham Math1^Δint^				
S.No.	Gene_ID	Mgi_Symbol	log2 Fold Change	*p*-adj
1	ENSMUSG00000096499	*Ighv1-5*	1.813	0.002

**Table 2:** Differentially expressed genes detected in the comparison of PPVL control vs. PPVL Math1^Δint^.

PPVL Control vs. PPVL Math1^Δint^				
S.No.	Gene_ID	Mgi_Symbol	log2 Fold Change	*p*-adj
1	ENSMUSG00000020407	*Upp1*	1.356	0.007
2	ENSMUSG00000003355	*Fkbp11*	1.241	0.007
3	ENSMUSG00000030659	*Nucb2*	1.222	0.007
4	ENSMUSG00000053695	*Defb37*	−1.598	0.007
5	ENSMUSG00000025196	*Cpn1*	−1.669	0.009
6	ENSMUSG00000070427	*Il18bp*	1.480	0.010
7	ENSMUSG00000093394	*Gm20621*	−1.598	0.011
8	ENSMUSG00000000440	*Pparg*	−1.242	0.013
9	ENSMUSG00000040543	*Pitpnm3*	−1.317	0.015
10	ENSMUSG00000029417	*Cxcl9*	1.482	0.016
11	ENSMUSG00000061825	*Ces2c*	−1.147	0.018
12	ENSMUSG00000095981	*Ighv10-1*	1.518	0.020
13	ENSMUSG00000096727	*Psmb9*	1.195	0.023
14	ENSMUSG00000074934	*Grem1*	−0.802	0.026
15	ENSMUSG00000079116	*Gm15293*	1.321	0.026
16	ENSMUSG00000081769	*Gm12216*	1.080	0.026
17	ENSMUSG00000004933	*Matk*	−1.476	0.026
18	ENSMUSG00000027514	*Zbp1*	1.467	0.026
19	ENSMUSG00000079339	*Ifit1bl1*	1.476	0.026
20	ENSMUSG00000055748	*Gsdmc4*	−1.433	0.026
21	ENSMUSG00000047844	*Bex4*	−1.457	0.026
22	ENSMUSG00000110639	*Gm6831*	−1.460	0.026
23	ENSMUSG00000067599	*Klra7*	1.428	0.026
24	ENSMUSG00000032418	*Me1*	−1.458	0.026
25	ENSMUSG00000094662	*Defa36*	1.334	0.026
26	ENSMUSG00000022216	*Psme1*	0.700	0.026
27	ENSMUSG00000076633	*Ighv5-2*	−1.433	0.027
28	ENSMUSG00000035692	*Isg15*	1.421	0.027
29	ENSMUSG00000056293	*Gsdmc2*	−1.374	0.027
30	ENSMUSG00000040808	*S100g*	−1.354	0.027
31	ENSMUSG00000046718	*Bst2*	0.892	0.027
32	ENSMUSG00000068614	*Actc1*	−1.414	0.030
33	ENSMUSG00000022964	*Tmem50b*	0.447	0.030
34	ENSMUSG00000095328	*Defa-ps6*	1.422	0.031
35	ENSMUSG00000035105	*Egln3*	−1.316	0.031
36	ENSMUSG00000094797	*Igkv6-15*	1.381	0.031
37	ENSMUSG00000021730	*Hcn1*	−1.415	0.031
38	ENSMUSG00000078612	*Fyb2*	−1.370	0.034
39	ENSMUSG00000055827	*Gsdmc3*	−1.336	0.034
40	ENSMUSG00000076512	*Igkv9-123*	1.225	0.034
41	ENSMUSG00000039323	*Igfbp2*	−1.388	0.036
42	ENSMUSG00000022587	*Ly6e*	0.953	0.036
43	ENSMUSG00000020469	*Myl7*	0.872	0.036
44	ENSMUSG00000025041	*Nt5c2*	−0.953	0.036
45	ENSMUSG00000026104	*Stat1*	1.133	0.036
46	ENSMUSG00000030107	*Usp18*	1.304	0.036
47	ENSMUSG00000098557	*Kctd12*	−1.194	0.037
48	ENSMUSG00000094687	*Defa25*	1.231	0.037
49	ENSMUSG00000078853	*Igtp*	1.372	0.037
50	ENSMUSG00000028965	*Tnfrsf9*	1.239	0.037
51	ENSMUSG00000052373	*Mpp3*	−1.332	0.038
52	ENSMUSG00000095565	*Ighv2-9-1*	1.294	0.041
53	ENSMUSG00000027397	*Slc20a1*	−1.194	0.043
54	ENSMUSG00000030173	*Klra5*	1.349	0.043
55	ENSMUSG00000018211	*Wfdc15b*	−1.296	0.043
56	ENSMUSG00000026981	*Il1rn*	−1.278	0.046
57	ENSMUSG00000050097	*Ces2b*	−1.344	0.046
58	ENSMUSG00000020319	*Wdpcp*	1.168	0.046
59	ENSMUSG00000024600	*Slc27a6*	−1.306	0.048
60	ENSMUSG00000054169	*Ceacam10*	1.288	0.048
61	ENSMUSG00000046460	*Sh2d7*	−1.333	0.049
62	ENSMUSG00000029307	*Dmp1*	−1.330	0.049
63	ENSMUSG00000020571	*Pdia6*	0.609	0.049

**Table 3:** Major biological processes enriched for differentially expressed genes in the comparison of PPVL control vs. PPVL Math1^Δintestine^. Annotated = total number of genes in GO term; Significant = total number of differentially expressed genes in term (*p*-adj < 0.05).

GO.ID	Term	Annotated	Significant	Weight01.Fisher *p*-Value	Genes
GO:0071222	Cellular response to lipopolysaccharide	177	5	0.00	*Cxcl9*, *Defa25*, *Gm15293*, *Il1rn*, *Stat1*
GO:0061844	Antimicrobial humoral immune response mediated by antimicrobial peptide	56	3	0.00	*Cxcl9*, *Defa25*, *Gm15293*
GO:0042742	Defense response to bacterium	185	5	0.00	*Defa25*, *Defb37*, *Gm15293*, *Isg15*, *Wfdc15b*
GO:0046321	Positive regulation of fatty acid oxidation	17	2	0.00	*Nucb2*, *Pparg*
GO:0045648	Positive regulation of erythrocyte differentiation	23	2	0.00	*Isg15*, *Stat1*
GO:0051673	Membrane disruption in other organism	25	2	0.00	*Defa25*, *Gm15293*
GO:0002227	Innate immune response in mucosa	31	2	0.00	*Defa25*, *Gm15293*
GO:0072608	Interleukin-10 secretion	12	2	0.01	*Isg15*, *Tnfrsf9*
GO:0061326	Renal tubule development	80	2	0.01	*Grem1*, *Stat1*
GO:0042127	Regulation of cell proliferation	1393	8	0.01	*Bex4*, *Egln3*, *Grem1*, *Igfbp2*, *Matk*, *Pparg*, *Stat1*, *Tnfrsf9*
GO:0019731	Antibacterial humoral response	38	2	0.01	*Defa25*, *Gm15293*
GO:0035458	Cellular response to interferon-beta	45	2	0.01	*Igtp*, *Stat1*
GO:0010742	Macrophage derived foam cell differentiation	23	2	0.01	*Pparg*, *Stat1*
GO:1900077	Negative regulation of cellular response to insulin stimulus	40	2	0.01	*Nucb2*, *Pparg*
GO:0090278	Negative regulation of peptide hormone secretion	56	2	0.02	*Bst2*, *Isg15*
GO:0051893	Regulation of focal adhesion assembly	56	2	0.02	*Grem1*, *Wdpcp*
GO:0034340	Response to type I interferon	37	3	0.02	*Isg15*, *Stat1*, *Zbp1*

## Data Availability

The data that support the findings of this study are available within the article and upon reasonable request to the corresponding author, S.M.

## Supplementary Materials

The following supporting information can be downloaded at: https://www.mdpi.com/article/10.3390/biomedicines10071503/s1. Figure S1: Manual quantitation of tubularization; Figure S2: LECs proliferation after treatment with organoids CM; Table S1: Primers used for genotyping of Math1^Lox/Lox^VilCreER^T2^ mice; Table S2: Details of probes used for TaqMan^®^ gene expression assays; Table S3: Proteins identified in secretome analysis of CM collected from organoids treated with LPS and MDP (Math1^Δint^ vs control).

## Abbreviations

Paneth cells (PCs), partial portal vein ligation (PPVL), immunohistochemistry (IHC), lymphatic vessel endothelial hyaluronic acid receptor 1 (LYVE1), pathogen-associated molecular patterns (PAMPs), conditioned media (CM), lymphatic microvascular endothelial cells (LECs), prospero-related homeobox 1 (*PROX1*), vascular endothelial growth factor C (VEGFC), vascular endothelial growth factor receptor 3 (*VEGFR3*), chronic liver disease (CLD), regenerating islet derived protein 3 (*REG3A*), endothelial nitric oxid synthase (eNOS), specific pathogen free (SPF), forkhead box C2 (*FOXC2*), 3,3-diaminobenzidine-tetrahydrochloride (DAB), ethylene diamine tetra acetic acid (EDTA), diamidino-2-phenylindole (DAPI), mass spectrometry (MS), lipopolysaccharide (LPS), muramyl dipeptide (MDP), peptidoglycan (PGN), CpG oligodeoxynucleotides (CpG ODN).
