# Supplementary material for: Paneth Cells Regulate Lymphangiogenesis under Control of Microbial Signals during Experimental Portal Hypertension
Source: Biomedicines. 2022 Jun 25;10(7):1503. doi: 10.3390/biomedicines10071503 (PMC9313283; doi:10.3390/biomedicines10071503)
Supplement: Supplementary file 1 [file biomedicines-10-01503-s001.zip › biomedicines-1702320-supplementary.pdf]

Title:

Paneth cells regulate lymphangiogenesis under control of microbial signals during experimental portal hypertension.

Authors:

Mohsin Hassan [1,2,†], Oriol Juanola [3,4,†], Irene Keller [5], Paolo Nanni [6], Witold Wolski [6], Sebastián Martínez-López [7,8], Esther Caparrós [7,8], Rubén Francés [7,8,9,10] and Sheida Moghadamrad [2,3,4,11,*]

Affiliations:

[1] Department of Hepatology & Gastroenterology, Charité Universitätsmedizin Berlin, 13353 Berlin, Germany; mohsin.hassan@charite.de (M.H.)

[2] Department for Biomedical Research (DBMR), University of Bern, University Clinic of Visceral Surgery and Medicine, Inselspital, Bern, Switzerland, 3008 Bern, Switzerland

[3] Laboratories for Translational Research, Department of Gastroenterology and Hepatology,

Ente Ospedaliero Cantonale, 6500 Bellinzona, Switzerland;

oriol.juanolajuarez@eoc.ch (O.J); sheida.moghadamrad@usi.ch (S.M)

[4] Faculty of Biomedical Sciences, Università della Svizzera Italiana, 6900 Lugano, Switzerland

[5] Interfaculty Bioinformatics Unit, Swiss Institute of Bioinformatics, University of Bern, 3008 Bern, Switzerland; i.keller@nexus.ethz.ch (I.K.)

[6] Functional Genomics Center Zurich, University/ETH Zurich, 8057 Zurich, Switzerland;

paolo.nanni@fgcz.uzh.ch (P.N.); wew.fgcz@gmail.com (W.W.)

[7] Hepatic and Intestinal Immunobiology Group, Departamento Medicina Clínica, Universidad Miguel Hernández, 03550 Alicante, Spain; sebastian.martinez@goumh.umh.es (S.M.-L.); ecaparros@umh.es (E.C.); rfrances@umh.es (R.F.)

[8] Instituto de Investigación Sanitaria ISABIAL, Hospital General Universitario, 03010 Alicante, Spain

[9] Instituto de Investigación, Desarrollo e Innovación en Biotecnología Sanitaria de Elche (IDiBE), Universidad Miguel Hernández, 03207 Elche, Spain

[10] CIBERehd, Instituto Salud Carlos III, 28029 Madrid, Spain

[11] Department of Hepatology, University Clinic of Visceral Surgery and Medicine, Inselspital, 3008 Bern, Switzerland

* Correspondence: sheida.moghadamrad@usi.ch; Tel.: +41-58-666-7117

† These authors contributed equally to this work.

Correspondence:

Dr.phil.nat. Sheida Moghadamrad

Translational Research Laboratory, Department of Gastroenterology and Hepatology, Ente Ospedaliero Cantonale, Lugano, Switzerland;

Università della Svizzera Italiana, Faculty of Biomedical Sciences, Lugano, Switzerland,

Hepatology Laboratory Research, Bellinzona Institutes of Science,

Ente Ospedaliero Cantonale, Via F. Chiesa 5, 6500 Bellinzona, Switzerland

Phone: +41 91 8210070

E-mail: sheida.moghadamrad@usi.ch

Supplementary Figure S1

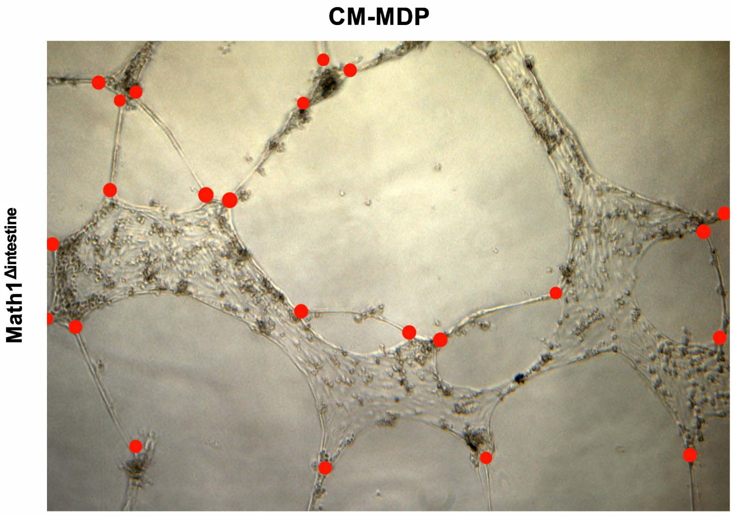

Supplementary Figure S1. Manual quantitation of tubularization. Clearly defined tube nodes were counted manually on each micrograph.

Supplementary Figure S2

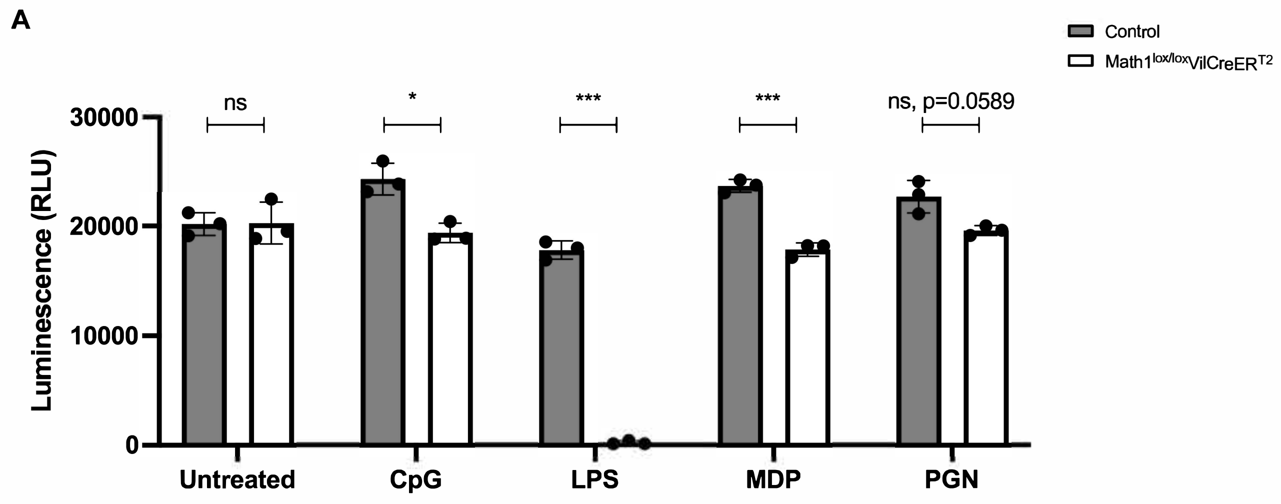

Supplementary Figure S2. LECs proliferation after treatment with organoids CM. (A) CellTiter-Glo® assay using $2x10^3$ LECs incubated with organoids CM for 12h. (B) CCK-8 assay using $2x10^3$ LECs

incubated with organoids CM for 12h. Abbreviations: CM, Conditioned media; CpG, CpG motifs of bacterial DNA; LECs, Lymphatic endothelial cells; LPS, Lipopolysaccharide; Math1$^{lox/lox}$VilCreER^{T2}, Paneth cell depleted; MDP, Muramyl di-peptide; ns, not significant; PGN, peptidoglycan; PCs, Paneth cells. Data are expressed as mean ± SD and is representative of 3 independent experiments. *p <0.05; **p <0.005; ***p <0.001.

Supplementary materials and methods

Sample preparation for proteomics

For each sample the protein concentration was estimated using the Qubit® Protein Assay Kit (Life Technologies, Zurich, Switzerland). The samples were then prepared by using a commercial iST Kit (PreOmics, Germany) with an updated version of the protocol. Briefly, 50µg of protein were solubilized in 'Lyse' buffer, boiled at 95°C for 10 minutes and processed with HIFU for 30seconds setting the ultrasonic amplitude to 85%. Afterwards, the samples were transferred to the cartridge and digested by adding 50µl of the 'Digest' solution. After 60minutes of incubation at 37°C the digestion was stopped with 100µl of Stop solution. The solutions in the cartridge were removed by centrifugation at 3800g, while the peptides were retained by the iST-filter. Finally, the peptides werewashed, eluted, dried and re-solubilized in40 µL of buffer (3% acetonitrile, 0.1% formic acid) buffer for LC-MS-Analysis.iRT retention time standard peptides (Biognosys, Switzerland) were added to each sample as of manufacturer instructions.

Liquid chromatography-mass spectrometry analysis

Mass spectrometry analysis was performed on a QExactive HF-X mass spectrometer (Thermo Scientific) equipped with a Digital PicoView source (New Objective) and coupled to a M-Class UPLC (Waters). Solvent composition at the two channels was 0.1% formic acid for channel A and 0.1% formic acid, 99.9% acetonitrile for channel B. For each sample 2µL of peptides were loaded on a commercial MZ Symmetry C18 Trap Column (100Å, 5 µm, 180 µm x 20 mm, Waters) followed by nanoEase MZ C18 HSS T3 Column (100Å, 1.8 µm, 75 µm x 250 mm, Waters). The peptides were eluted

at a flow rate of 300 nL/min by a gradient from 8 to 27% B in 82 min, 35% B in 5 min and 80% B in 1 min. Samples were acquired in a randomized order. The mass spectrometer was operated in data-dependent mode (DDA), acquiring a full-scan MS spectrum (350−1’400 m/z) at a resolution of 120’000 at 200 m/z after accumulation to a target value of 3’000’000, followed by HCD (higher-energy collision dissociation) fragmentation on the twenty most intense signals per cycle. HCD spectra were acquired at a resolution of 15’000 using a normalized collision energy of 28 and a maximum injection time of 22ms. The automatic gain control (AGC) was set to 100’000 ions. Charge state screening was enabled. Singly, unassigned, and charge states higher than seven were rejected. Only precursors with intensity above 110’000 were selected for MS/MS. Precursor masses previously selected for MS/MS measurement were excluded from further selection for 30 s, and the exclusión window was set at 10 ppm. The samples were acquired using internal lock mass calibration on m/z 371.1012 and 445.1200.

The mass spectrometry proteomics data were handled using the local laboratory information management system (LIMS).

Protein identification and label free protein quantification

The acquired raw MS data were processed by MaxQuant (version 1.6.2.3), followed by protein identification using the integrated Andromeda search engine. Spectra were searched against a Uniprot mouse reference proteome (taxonomy 10090, canonical version from 2016-09-02), concatenated to its reversed decoyed fasta database and common protein contaminants. Carbamidomethylation of cysteine was set as fixed modification, while methionine oxidation and N-terminal protein acetylation were set

as variable. Enzyme specificity was set to trypsin/P allowing a minimal peptide length of 7 amino acids and a maximum of two missed-cleavages. MaxQuant Orbitrap default search settings were used. The maximum false discovery rate (FDR) was set to 0.01 for peptides and 0.05 for proteins. Label free quantification was enabled and a 2 minutes window for match between runs was applied. In the MaxQuant experimental design template, each file is kept separate in the experimental design to obtain individual quantitative values. Protein fold changes were computed based on Intensity values reported in the proteinGroups.txt file. A set of functions implemented in the R package SRMService was used to filter for proteins with 2 or more peptides allowing for a maximum of 4 missing values, and to normalize the data with a modified robust z-score transformation and to compute p-values using the t-test with pooled variance. If all measurements of a protein are missing in one of the conditions, a pseudo fold change was computed replacing the missing group average by the mean of 10% smallest protein intensities in that condition.

Cell proliferation assay

LECs at passage 8 were serum and growth factors starved overnight. A total of $2x10^3$/well LECs were cultured in a 96-well plate and incubated with organoids CM in a total volume of 100µL for 12 hours at 37°C, 5% CO_2. Cell proliferative capacity of LECs was measured by two methods: CellTiter-Glo® (Promega) and Cell counting Kit-8 (Dojindo) in independent experiments. In the first, 100µL of CellTiter-Glo® reagent was added to the wells and the 96-well plate was incubated for 2 minutes in an orbital shaker to induce cell lysis. Luminiscence was then recorded in the Infinite M Plex plate reader (Tecan). In the second assay, 10µL of CCK-8 solution were added to each

experimental well and the plate was incubated for 4 hours in the incubator at 37°C, 5% CO_2. Then, absorbance at 450 nm was measured using the Infinite M Plex plate reader (Tecan) using a wavelength correction at 600 nm. In both experiments we included control wells containing medium without cells to obtain values for background luminescence and absorbance.

Supplementary table S1: Primers used for genotyping of Math1$^{Lox/Lox}$VilCreER^{T2} mice.

S.No.	Primer	Sequence
1	Cre-F	5’-CCTGGAAAATGCTTCTGTCCG-3’
2	Cre-R	5’-CAGGGTGTTATAAGCAATCCC-3’
3	Math1Lox-F	5’-CAGATCCCACAGAAGTGACG-3’
4	Math1Lox-R-	5’-ACACTGCTGGACACACTTGG-3’

Supplementary table S2: Details of probes used for TaqMan® gene expression assays.

S.No.	Reagent	Dilution	Source	Identifier
1	FOXC-2	1:20	ThermoFisher Scientific	Mm00546194_s1
2	PROX-1	1:20	ThermoFisher Scientific	Mm00435969_m1
3	VEGFR2	1:20	ThermoFisher Scientific	Mm01222421_m1
4	VEGFR3	1:20	ThermoFisher Scientific	Mm01292604_m1

Supplementary table S3: Proteins identified in secretome analysis of CM collected from organoids treated with LPS and MDP (Math1$^{\Delta int}$ vs control).

LPS-treated organoids (Control vs Math1Δint)			
**Protein_ID**	**Protein name**	**log2FoldChange**	**Adj. *p*-value**
Q06890	CLUS	-3,066	0,001
Q8R0I0	ACE2	3,261	0,002
P84244	H33	-1,627	0,004
Q9R1P1	PSB3	1,346	0,004
Q9DCD0	6PGD	-1,553	0,006
O88968	TCO2	2,254	0,006
P62806	H4	-1,390	0,006
O35658	C1QBP	1,298	0,006
Q9R1P3	PSB2	1,031	0,007
P43137	LIT1	1,950	0,007
P24549	AL1A1	-1,119	0,008
Q07797	LG3BP	1,065	0,008
P16110	LEG3	-1,953	0,008
Q922R8	PDIA6	1,557	0,008
P16014	SCG1	1,874	0,011
P48758	CBR1	-1,223	0,012
P10493	NID1	-2,004	0,012
Q02257	PLAK	-1,854	0,012
P19137	LAMA1	-2,096	0,012
P14869	RLA0	-1,118	0,012
Q8K0C5	ZG16	1,244	0,012
Q8K0C9	GMDS	-2,371	0,013
P02469	LAMB1	-2,010	0,013
P16858	G3P	-1,163	0,013
Q9EQF5	DPYS	-1,116	0,013
O88569	ROA2	-0,989	0,013
P10126	EF1A1	-0,971	0,013
P02468	LAMC1	-1,966	0,013
P29391	FRIL1	0,912	0,013
Q02819	NUCB1	0,847	0,014
Q9R1P0	PSA4	1,267	0,014
Q9QXD6	F16P1	3,050	0,014
Q9CZU6	CISY	-1,141	0,015
P15864	H12	-1,518	0,015
P63038	CH60	0,859	0,016
P09411	PGK1	-0,747	0,020

Q8BK48	EST2E	0,911	0,020
ZZ_FGCZCont0050	ZZ_FGCZCont0050	-1,028	0,021
O35945	AL1A7	-0,968	0,021
O08749	DLDH	1,718	0,021
P63017	HSP7C	-0,788	0,022
Q80W65	PCSK9	0,947	0,022
Q3UZZ4	OLFM4	-1,056	0,022
P68373	TBA1C	-2,636	0,024
P52480	KPYM	-0,834	0,024
Q9Z2U1	PSA5	1,328	0,024
P63260	ACTG	-0,817	0,026
O09131	GSTO1	-0,777	0,026
P68510	1433F	-0,717	0,026
Q8VC30	TKFC	-1,351	0,027
P32020	NLTP	1,333	0,027
P24822	PPBI	-0,683	0,027
P13745	GSTA1	-0,685	0,029
P97449	AMPN	0,832	0,029
Q9CQI6	COTL1	2,067	0,029
P38647	GRP75	0,882	0,029
P16546	SPTN1	1,533	0,029
P01898	HA10	2,291	0,029
P06728	APOA4	0,838	0,030
Q80XD8	PRAP1	0,660	0,032
O09051	GUC2B	2,274	0,033
P12710	FABPL	-0,697	0,034
O08585	CLCA	1,462	0,035
P68372	TBB4B	-1,616	0,035
Q00623	APOA1	0,935	0,037
P09671	SODM	1,043	0,037
P19639	GSTM4	-1,924	0,040
P23492	PNPH	2,156	0,042
P11499	HS90B	-0,837	0,045
P35979	RL12	-1,253	0,045
P00329	ADH1	-0,671	0,047
Q91WU0	CES1F	-1,669	0,049
P30275	KCRU	-0,973	0,055
P10649	GSTM1	-0,581	0,055
Q05793	PGBM	-1,043	0,055
P19157	GSTP1	-0,996	0,057
Q8CGP6	H2A1H	-0,634	0,057
Q9D8N0	EF1G	-1,546	0,057

P24270	CATA	-0,730	0,057
P17742	PPIA	-0,623	0,057
O88322	NID2	-1,663	0,057
P10605	CATB	1,159	0,057
P47738	ALDH2	-1,063	0,062
P09405	NUCL	-1,253	0,063
P97429	ANXA4	0,644	0,064
Q99PT1	GDIR1	1,515	0,065
P00493	HPRT	1,648	0,066
A2ASS6	TITIN	-1,476	0,070
Q8K157	GALM	0,756	0,077
P99026	PSB4	0,827	0,081
P07356	ANXA2	0,585	0,083
O70570	PIGR	0,536	0,084
Q9D6J6	NDUV2	0,512	0,090
Q64133	AOFA	0,904	0,094
P53657	KPYR	-1,309	0,094
P20029	GRP78	0,477	0,096
Q61171	PRDX2	0,494	0,098
Q9Z2U0	PSA7	0,636	0,098
Q99KP3	CRYL1	1,002	0,098
Q6ZWM4	LSM8	0,706	0,099
Q9WTP6	KAD2	1,178	0,099
O70435	PSA3	0,599	0,100
Q99LX0	PARK7	0,768	0,100
Q9WUM4	COR1C	0,999	0,100
Q9D312	K1C20	0,846	0,102
P50247	SAHH	-0,563	0,109
P47754	CAZA2	-1,002	0,114
Q9EQK5	MVP	-0,891	0,117
Q6URW6	MYH14	0,792	0,127
P14206	RSSA	-1,418	0,132
P58252	EF2	-0,540	0,137
Q9R0P3	ESTD	-1,269	0,149
P17563	SBP1	-0,501	0,149
P11725	OTC	0,789	0,155
Q9QXT0	CNPY2	0,882	0,155
P05201	AATC	2,112	0,166
Q60864	STIP1	-0,615	0,166
P84089	ERH	-0,819	0,168
P62962	PROF1	-0,476	0,168
Q60605	MYL6	0,488	0,171

Q91YR9	PTGR1	0,620	0,171
P62827	RAN	-1,061	0,176
O88342	WDR1	-0,787	0,176
P40142	TKT	-0,462	0,176
Q9CZ44	NSF1C	0,605	0,176
P49722	PSA2	0,537	0,184
Q9R100	CAD17	-0,541	0,194
Q8K354	CBR3	-0,498	0,199
Q62433	NDRG1	-0,517	0,203
Q9CQV8	1433B	-1,199	0,205
P05784	K1C18	-0,411	0,208
P27773	PDIA3	0,356	0,211
P68134	ACTS	-1,020	0,211
Q8R1G2	CMBL	0,714	0,211
Q9EPB4	ASC	0,486	0,214
Q8CAQ8	MIC60	0,843	0,215
P18760	COF1	-0,399	0,216
Q8K0E8	FIBB	-2,558	0,227
Q920E5	FPPS	-0,374	0,227
Q9D1A2	CNDP2	0,863	0,227
Q60692	PSB6	0,981	0,227
Q9JLJ2	AL9A1	-0,758	0,227
P08003	PDIA4	1,133	0,230
P55050	FABPI	0,556	0,247
P13707	GPDA	-0,445	0,252
P70168	IMB1	-0,387	0,252
P35700	PRDX1	0,372	0,253
P08228	SODC	0,387	0,253
P01887	B2MG	0,602	0,253
P56395	CYB5	-0,553	0,254
Q9D154	ILEUA	0,707	0,254
P63242	IF5A1	-0,718	0,255
Q61508	ECM1	-0,524	0,255
P62204	CALM	0,524	0,255
O88310	ITL1A	-0,461	0,257
O88312	AGR2	-0,323	0,257
Q9R097	SPIT1	0,874	0,257
P68037	UB2L3	0,525	0,266
Q8C196	CPSM	0,288	0,277
Q9R0P5	DEST	-0,337	0,283
P39061	COIA1	-1,045	0,289
P19467	MUC13	0,307	0,296

Q0VG18	SIM24	0,926	0,305
P97371	PSME1	0,356	0,309
P40124	CAP1	-0,646	0,316
Q62468	VILI	-0,512	0,316
Q91WG0	EST2C	-0,391	0,316
Q04447	KCRB	-0,315	0,316
Q8K2B3	SDHA	-0,946	0,323
P05202	AATM	-0,664	0,323
Q8C115	PKHH2	-0,431	0,323
P99029	PRDX5	0,649	0,323
Q60997	DMBT1	0,366	0,323
P09528	FRIH	0,559	0,324
P20108	PRDX3	0,718	0,330
O89079	COPE	-0,335	0,333
P34884	MIF	-0,652	0,333
Q61425	HCDH	0,636	0,333
P51855	GSHB	0,898	0,333
Q8K419	LEG4	-0,280	0,335
P21107	TPM3	1,110	0,349
Q08652	RET2	-0,568	0,352
Q08879	FBLN1	-0,335	0,352
Q9WVA4	TAGL2	0,322	0,352
P97807	FUMH	0,698	0,352
P24527	LKHA4	-0,440	0,352
Q9Z0S1	BPNT1	0,303	0,356
E9Q557	DESP	-0,973	0,362
O70475	UGDH	-1,500	0,369
Q61937	NPM	-0,418	0,369
P17751	TPIS	-0,361	0,370
Q91Y97	ALDOB	-0,275	0,377
P26040	EZRI	0,279	0,377
Q8R0W0	EPIPL	0,475	0,377
Q8VDN2	AT1A1	-0,587	0,386
P24472	GSTA4	-0,315	0,389
P17182	ENOA	-0,307	0,389
Q9CQW5	LEG2	-0,223	0,389
Q91YR1	TWF1	0,691	0,389
P60843	IF4A1	-1,017	0,402
Q6P069	SORCN	-0,386	0,407
Q62393	TPD52	-0,730	0,411
Q8R0F8	FAHD1	1,208	0,417
P99027	RLA2	-0,557	0,417

P12023	A4	-0,388	0,417
O35459	ECH1	0,944	0,417
Q7TPR4	ACTN1	-2,176	0,430
P08122	CO4A2	-2,008	0,458
Q9QYX7	PCLO	-2,430	0,463
P16406	AMPE	0,257	0,464
P56480	ATPB	-0,322	0,471
P05064	ALDOA	-0,226	0,472
Q91X72	HEMO	-0,312	0,474
P08113	ENPL	-0,351	0,488
Q80X90	FLNB	0,319	0,502
P54071	IDHP	-0,576	0,521
Q61792	LASP1	-0,553	0,547
P49312	ROA1	-0,265	0,547
P14152	MDHC	-0,204	0,561
Q9QUM9	PSA6	0,180	0,561
P70441	NHRF1	0,323	0,561
P97816	S100G	-0,487	0,576
P06745	G6PI	-0,207	0,576
P17897	LYZ1	-0,244	0,577
P24369	PPIB	-0,410	0,591
P59999	ARPC4	-0,409	0,594
O70456	1433S	-0,205	0,595
Q8QZR3	EST2A	-0,572	0,606
Q8BND5	QSOX1	-0,424	0,606
Q9DBJ1	PGAM1	0,161	0,606
Q9JMH6	TRXR1	0,237	0,606
O55234	PSB5	0,578	0,611
O55111	DSG2	0,462	0,621
P10639	THIO	0,185	0,623
O08807	PRDX4	0,192	0,623
P42125	ECI1	0,247	0,623
P30681	HMGB2	-0,242	0,628
Q8BHN3	GANAB	0,203	0,637
Q60854	SPB6	0,541	0,645
P68254	1433T	-0,446	0,654
P70195	PSB7	0,368	0,669
Q01768	NDKB	0,207	0,673
P63101	1433Z	-0,161	0,682
P31786	ACBP	0,157	0,682
Q8BG05	ROA3	0,206	0,686
Q8VC28	AK1CD	-0,196	0,687

Q9R1P4	PSA1	0,178	0,700
Q9Z0L8	GGH	0,173	0,702
Q9JKF1	IQGA1	-0,268	0,703
Q9DCV7	K2C7	0,192	0,703
P47968	RPIA	0,218	0,703
Q3V0K9	PLSI	0,319	0,703
Q91YI0	ARLY	0,159	0,704
P48756	GIP	-0,200	0,704
P57780	ACTN4	0,120	0,704
P61089	UBE2N	-0,255	0,708
Q8CHP8	PGP	0,174	0,708
Q9D0F9	PGM1	0,304	0,757
Q3URE1	ACSF3	-0,203	0,758
Q9QUR6	PPCE	0,163	0,761
Q9CPU0	LGUL	-0,199	0,773
Q61598	GDIB	0,086	0,782
Q99JW5	EPCAM	-0,143	0,783
Q9Z2X1	HNRPF	-0,254	0,788
Q99020	ROAA	0,546	0,788
Q99104	MYO5A	-0,080	0,790
P09103	PDIA1	-0,088	0,806
Q9EPL2	CSTN1	-0,187	0,812
P29758	OAT	0,078	0,812
Q91ZJ5	UGPA	0,080	0,812
Q9EQU5	SET	-0,117	0,839
Q91WL0	ES8L3	0,089	0,839
O88844	IDHC	-0,063	0,845
P47791	GSHR	-0,176	0,849
Q9CQA3	SDHB	-0,156	0,849
P19001	K1C19	0,080	0,849
P06151	LDHA	0,059	0,859
Q9CPT4	MYDGF	0,167	0,859
P06801	MAOX	0,291	0,859
Q05816	FABP5	-0,116	0,868
Q62261	SPTB2	-0,087	0,868
P21460	CYTC	0,063	0,892
Q01853	TERA	0,098	0,892
Q9D819	IPYR	0,107	0,892
Q8VDD5	MYH9	-0,089	0,896
P07901	HS90A	-0,117	0,897
Q7TPH6	MYCB2	-0,094	0,897
P61458	PHS	0,064	0,904

O35490	BHMT1	-0,078	0,915
O88531	PPT1	-0,085	0,916
P53810	PIPNA	0,118	0,916
Q9JM76	ARPC3	-0,050	0,916
Q64433	CH10	-0,058	0,921
P11679	K2C8	-0,033	0,922
P14211	CALR	0,034	0,922
Q8BWT1	THIM	-0,052	0,925
P26369	U2AF2	0,122	0,925
Q62165	DAG1	-0,071	0,939
Q99KI0	ACON	-0,029	0,951
P26443	DHE3	0,023	0,951
P70296	PEBP1	0,035	0,951
O35639	ANXA3	0,052	0,951
P97466	NOGG	0,064	0,951
P47757	CAPZB	-0,047	0,954
Q8R180	ERO1A	-0,036	0,960
Q03265	ATPA	-0,054	0,960
Q5SV42	ILEUC	0,020	0,960
P48678	LMNA	-0,025	0,963
Q93092	TALDO	0,023	0,981
E9PZQ0	RYR1	-0,020	0,985
Q6ZQ06	CE162	-0,012	0,993
P62259	1433E	-0,004	0,993
P08249	MDHM	-0,004	0,993
P30416	FKBP4	-0,004	0,993

**MDP-treated organoids (Control vs Math1Δint)**			
**Protein_ID**	**Protein name**	**log2FoldChange**	**Adj. *p*-value**
P17563	SBP1	-2,296	0,000
Q8R0I0	ACE2	3,056	0,000
P43137	LIT1	4,233	0,001
Q9QXD6	F16P1	4,461	0,002
P12023	A4	-2,370	0,003
Q06890	CLUS	-1,944	0,003
P09411	PGK1	-1,626	0,003
P17897	LYZ1	-1,413	0,003
Q8BND5	QSOX1	-2,224	0,004
P62806	H4	-1,648	0,004
P97449	AMPN	1,332	0,004
P16406	AMPE	1,412	0,004

O09051	GUC2B	2,730	0,004
Q60997	DMBT1	1,299	0,004
P11499	HS90B	-1,494	0,004
Q8R180	ERO1A	-1,630	0,004
P12710	FABPL	1,034	0,005
O88968	TCO2	1,307	0,005
P13707	GPDA	1,379	0,007
P52480	KPYM	-1,137	0,009
P06728	APOA4	1,037	0,009
P84244	H33	-1,582	0,012
P10493	NID1	-1,306	0,012
P02469	LAMB1	-1,225	0,012
P17742	PPIA	-1,075	0,012
P40142	TKT	-1,058	0,012
Q60605	MYL6	1,218	0,012
P02468	LAMC1	-1,250	0,012
P09803	CADH1	-0,932	0,012
O08585	CLCA	1,516	0,012
O55111	DSG2	-1,509	0,013
P09405	NUCL	-1,017	0,014
P06745	G6PI	-1,013	0,014
P05064	ALDOA	-0,955	0,014
Q93092	TALDO	-0,908	0,014
P19137	LAMA1	-1,345	0,015
Q99020	ROAA	-3,222	0,015
Q9EPL2	CSTN1	-1,531	0,015
O88342	WDR1	-0,925	0,015
Q8K0C9	GMDS	-0,897	0,015
P58044	IDI1	-1,597	0,016
Q8K354	CBR3	1,836	0,017
Q920E5	FPPS	-1,216	0,017
P84089	ERH	-1,262	0,018
Q0VG18	SIM24	2,409	0,018
P24822	PPBI	1,363	0,019
Q9R100	CAD17	-0,970	0,020
Q00623	APOA1	1,257	0,020
Q99JG3	ANX13	0,973	0,020
Q99KP3	CRYL1	0,990	0,021
Q8CGP6	H2A1H	-0,851	0,022
E9PZQ0	RYR1	0,752	0,025
Q8BK48	EST2E	0,774	0,028
P68373	TBA1C	-1,794	0,029

P62962	PROF1	-0,688	0,029
P50247	SAHH	-0,673	0,029
Q8C196	CPSM	-0,722	0,032
P06151	LDHA	-0,817	0,033
O88310	ITL1A	1,311	0,033
P68037	UB2L3	-0,875	0,035
Q9DBJ1	PGAM1	-0,713	0,035
P70168	IMB1	-1,085	0,035
Q8CAQ8	MIC60	1,023	0,037
P56395	CYB5	1,456	0,037
Q05793	PGBM	-0,678	0,037
P17751	TPIS	-0,812	0,038
P58252	EF2	-0,734	0,038
Q9D8I3	GLOD5	1,338	0,038
Q80X90	FLNB	-0,846	0,040
P29758	OAT	-0,770	0,040
P21460	CYTC	-0,577	0,040
Q8R1G2	CMBL	0,887	0,040
Q61598	GDIB	-0,626	0,044
P19467	MUC13	0,638	0,044
P62858	RS28	-1,334	0,044
Q9QUR6	PPCE	-1,747	0,045
P17182	ENOA	-0,727	0,053
P70296	PEBP1	-0,665	0,053
P16110	LEG3	-1,081	0,061
Q99KI0	ACON	-0,855	0,062
Q8VCN5	CGL	1,113	0,065
P63017	HSP7C	-0,612	0,070
Q9WVA4	TAGL2	-0,492	0,077
P34884	MIF	-0,831	0,078
Q03265	ATPA	0,890	0,078
Q64133	AOFA	0,951	0,078
P53810	PIPNA	1,008	0,080
P10649	GSTM1	-0,567	0,082
Q61171	PRDX2	-0,566	0,082
O35658	C1QBP	0,678	0,085
Q9D154	ILEUA	0,583	0,086
Q08652	RET2	1,334	0,086
O88844	IDHC	-0,517	0,092
P97371	PSME1	0,633	0,097
Q9CZU6	CISY	-0,712	0,097
Q8C115	PKHH2	-0,509	0,105

Q9JKF1	IQGA1	-0,945	0,107
A2ASS6	TITIN	1,202	0,107
Q64433	CH10	-0,457	0,111
Q02819	NUCB1	0,574	0,111
O08709	PRDX6	-0,436	0,111
Q91WL0	ES8L3	0,963	0,113
Q08879	FBLN1	-0,560	0,113
Q9CQA3	SDHB	0,784	0,113
Q9QXT0	CNPY2	1,036	0,121
P61458	PHS	-0,825	0,121
P54071	IDHP	-0,977	0,129
Q9JM76	ARPC3	0,901	0,136
P23492	PNPH	0,688	0,142
Q9EQF5	DPYS	-0,682	0,144
Q01768	NDKB	-0,659	0,145
Q9D1A2	CNDP2	1,891	0,145
Q8VC30	TKFC	-1,118	0,148
Q8BWT1	THIM	-0,760	0,153
P18760	COF1	-0,384	0,154
P05202	AATM	-0,808	0,155
Q8K0C5	ZG16	0,592	0,157
Q4KML4	ABRAL	-0,746	0,157
P21107	TPM3	0,791	0,163
P14152	MDHC	-0,378	0,165
Q9D8N0	EF1G	-0,653	0,169
P23198	CBX3	-1,345	0,169
Q9EQK5	MVP	0,950	0,171
P38647	GRP75	-0,405	0,174
Q9WUM4	COR1C	-1,256	0,178
Q9R0P3	ESTD	-0,811	0,178
P01898	HA10	-0,805	0,180
P99026	PSB4	-0,436	0,182
P70441	NHRF1	0,445	0,191
P47738	ALDH2	-0,619	0,200
Q9R1P1	PSB3	0,695	0,200
Q9D6J6	NDUV2	0,997	0,200
Q60854	SPB6	0,897	0,216
Q91Y97	ALDOB	0,445	0,219
Q9JLJ2	AL9A1	-0,500	0,229
Q91YR9	PTGR1	0,897	0,233
Q9CQI6	COTL1	-0,683	0,237
Q02257	PLAK	0,927	0,237

Q9DCD0	6PGD	-0,948	0,244
Q62468	VILI	-0,746	0,244
P08228	SODC	-0,442	0,244
P27773	PDIA3	-0,364	0,244
E9Q557	DESP	0,607	0,244
P63101	1433Z	-0,329	0,250
Q91WG0	EST2C	0,673	0,254
Q6Q473	CLA4A	0,387	0,254
Q9QUM9	PSA6	0,817	0,256
Q6URW6	MYH14	0,523	0,266
Q91WU0	CES1F	0,709	0,266
Q9R1P3	PSB2	0,900	0,270
E9PV24	FIBA	-0,781	0,276
P48678	LMNA	0,539	0,277
Q8K0E8	FIBB	-0,875	0,286
P09671	SODM	0,510	0,288
P31786	ACBP	-0,303	0,289
Q99PT1	GDIR1	0,725	0,295
Q8BH95	ECHM	-0,845	0,297
Q9Z2U0	PSA7	0,355	0,301
P47754	CAZA2	-0,810	0,301
Q8BHN3	GANAB	-0,826	0,305
P07901	HS90A	-0,646	0,305
P24270	CATA	-0,424	0,305
P99029	PRDX5	-0,366	0,314
Q62261	SPTB2	-0,362	0,317
Q9D819	IPYR	-0,421	0,320
P07356	ANXA2	-0,356	0,324
O35945	AL1A7	-0,628	0,325
Q9Z2X1	HNRPF	-0,763	0,326
P20029	GRP78	-0,274	0,326
P08249	MDHM	-0,416	0,330
P10126	EF1A1	-0,312	0,333
O88312	AGR2	-0,266	0,341
P00329	ADH1	0,346	0,341
Q91ZJ5	UGPA	0,371	0,341
P62827	RAN	0,419	0,341
Q9R1P4	PSA1	0,446	0,341
P60843	IF4A1	0,828	0,341
P32020	NLTP	1,001	0,341
P97466	NOGG	-0,369	0,345
Q80XD8	PRAP1	0,262	0,360

P36552	HEM6	-0,776	0,362
Q99LX0	PARK7	-0,500	0,362
P63260	ACTG	-0,310	0,363
P99027	RLA2	-0,373	0,364
Q11136	PEPD	-1,296	0,379
P59999	ARPC4	-0,600	0,379
Q9CQV8	1433B	0,322	0,379
Q6P069	SORCN	0,328	0,379
P68134	ACTS	0,413	0,379
O08807	PRDX4	0,303	0,380
P08122	CO4A2	0,550	0,383
P24527	LKHA4	-0,649	0,392
Q8CHP8	PGP	-0,257	0,414
P24549	AL1A1	0,244	0,438
P19157	GSTP1	-0,243	0,443
P26369	U2AF2	-0,960	0,444
P05063	ALDOC	-0,553	0,444
Q9R0P5	DEST	-0,275	0,444
P10518	HEM2	0,657	0,444
P57780	ACTN4	-0,229	0,457
Q9CZ44	NSF1C	-0,497	0,470
Q9WV55	VAPA	-0,367	0,470
P09103	PDIA1	-0,219	0,470
Q6ZQ06	CE162	2,402	0,470
P99024	TBB5	-0,481	0,471
Q9JKR6	HYOU1	-0,365	0,473
Q8VC28	AK1CD	-0,197	0,473
P63038	CH60	0,266	0,473
Q7TPR4	ACTN1	1,963	0,482
Q9R1P0	PSA4	-0,431	0,487
Q91YI0	ARLY	-0,601	0,492
P05201	AATC	-0,607	0,497
Q8BG05	ROA3	0,424	0,497
Q5SV42	ILEUC	-0,271	0,497
P14869	RLA0	-0,227	0,497
Q8VCM7	FIBG	-0,541	0,510
P15864	H12	-0,601	0,511
Q8K157	GALM	0,449	0,519
P39061	COIA1	0,450	0,519
Q9CQW5	LEG2	0,272	0,522
P27467	WNT3A	0,368	0,522
P30681	HMGB2	0,374	0,522

P48758	CBR1	0,190	0,525
Q9Z0S1	BPNT1	0,293	0,528
P35979	RL12	-0,423	0,533
P10639	THIO	-0,190	0,544
P48756	GIP	0,341	0,544
P06801	MAOX	0,659	0,553
P51855	GSHB	-0,482	0,561
Q9JMH6	TRXR1	-0,195	0,561
P30275	KCRU	0,449	0,562
P97816	S100G	-0,383	0,572
O35639	ANXA3	-0,269	0,579
P63242	IF5A1	-0,372	0,584
Q07797	LG3BP	0,145	0,584
Q01853	TERA	0,315	0,584
P26443	DHE3	-0,175	0,585
Q61937	NPM	-0,151	0,585
Q8QZR3	EST2A	0,516	0,585
O09131	GSTO1	0,462	0,585
O35490	BHMT1	-0,386	0,590
Q9EQU5	SET	-0,275	0,590
P49312	ROA1	-0,253	0,590
Q91X72	HEMO	-0,188	0,590
Q9D312	K1C20	0,259	0,590
P70195	PSB7	0,288	0,590
Q91V76	CK054	0,559	0,590
Q9QYX7	PCLO	-1,490	0,603
P40124	CAP1	0,322	0,616
O89079	COPE	-0,155	0,621
P24472	GSTA4	-0,228	0,625
Q62433	NDRG1	-0,314	0,629
Q3URE1	ACSF3	-0,250	0,632
P62259	1433E	-0,138	0,632
Q61508	ECM1	0,140	0,632
Q8K2B3	SDHA	-0,566	0,644
P97807	FUMH	-0,367	0,660
O35459	ECH1	0,272	0,671
O08749	DLDH	0,226	0,675
O88569	ROA2	-0,140	0,675
P55292	DSC2	-0,331	0,677
P24369	PPIB	-0,155	0,686
P68254	1433T	0,242	0,687
P16014	SCG1	0,214	0,717

P56480	ATPB	-0,136	0,719
P35700	PRDX1	-0,132	0,719
P68510	1433F	0,127	0,747
Q3V0K9	PLSI	0,209	0,747
Q61792	LASP1	-0,254	0,762
O70475	UGDH	-0,280	0,766
P00493	HPRT	-0,356	0,768
O70456	1433S	-0,129	0,772
Q8VDD5	MYH9	0,112	0,807
P01887	B2MG	0,159	0,807
Q9CPT4	MYDGF	-0,102	0,824
Q9Z0L8	GGH	-0,087	0,824
P08113	ENPL	0,072	0,824
Q9R0H0	ACOX1	0,244	0,826
Q3UZZ4	OLFM4	-0,066	0,837
P47791	GSHR	0,184	0,837
O35685	NUDC	0,396	0,837
Q9CPU0	LGUL	0,103	0,840
Q60864	STIP1	-0,102	0,841
Q62165	DAG1	-0,101	0,841
O70435	PSA3	0,093	0,866
Q8VDN2	AT1A1	0,091	0,877
P14211	CALR	0,058	0,877
P16858	G3P	-0,074	0,893
P47757	CAPZB	-0,068	0,895
P97429	ANXA4	-0,053	0,895
P30416	FKBP4	0,080	0,895
Q60692	PSB6	0,104	0,895
Q9Z2U1	PSA5	0,060	0,902
Q8R0F8	FAHD1	0,254	0,904
P20108	PRDX3	0,045	0,907
Q99104	MYO5A	0,050	0,907
Q99JW5	EPCAM	0,096	0,913
Q04447	KCRB	-0,055	0,923
Q9EPB4	ASC	0,056	0,923
P19639	GSTM4	0,104	0,923
Q62393	TPD52	0,148	0,923
P11725	OTC	-0,046	0,931
P13745	GSTA1	-0,030	0,931
Q80W65	PCSK9	0,057	0,931
P68372	TBB4B	-0,059	0,932
P61089	UBE2N	-0,055	0,932

Q9WTP6	KAD2	-0,040	0,932
Q7TPH6	MYCB2	-0,051	0,934
P49722	PSA2	-0,046	0,934
P09528	FRIH	-0,036	0,934
P42125	ECI1	0,033	0,934
P19001	K1C19	0,038	0,934
Q61425	HCDH	0,046	0,934
Q64442	DHSO	0,066	0,934
O70570	PIGR	-0,024	0,937
P16546	SPTN1	-0,074	0,951
P10605	CATB	-0,043	0,951
Q8K419	LEG4	0,018	0,951
O88322	NID2	0,076	0,951
P47968	RPIA	0,034	0,957
Q9DCV7	K2C7	-0,024	0,959
P15626	GSTM2	-0,030	0,970
Q05816	FABP5	-0,023	0,970
Q6ZWM4	LSM8	-0,021	0,970
Q64727	VINC	-0,018	0,977
O88531	PPT1	0,019	0,979
P11679	K2C8	-0,007	0,981
P26040	EZRI	-0,007	0,985
P05784	K1C18	-0,002	0,993
P55050	FABPI	-0,001	0,997
